# Fetoplacental extracellular vesicles deliver conceptus-derived antigens to maternal secondary lymphoid tissues for immune recognition

**DOI:** 10.1172/jci.insight.186335

**Published:** 2025-05-22

**Authors:** Juliana S. Powell, Adriana T. Larregina, William J. Shufesky, Mara L.G. Sullivan, Donna Beer Stolz, Stephen J. Gould, Geoffrey Camirand, Sergio D. Catz, Simon C. Watkins, Yoel Sadovsky, Adrian E. Morelli

**Affiliations:** 1 T.E. Starzl Transplantation Institute, Department of Surgery;; 2 Department of Obstetrics, Gynecology, and Reproductive Sciences, Magee-Womens Research Institute;; 3 Department of Dermatology;; 4 Department of Immunology;; 5 McGowan Institute for Regenerative Medicine; and; 6 Department of Cell Biology, University of Pittsburgh, Pittsburgh, Pennsylvania, USA.; 7 Department of Biological Chemistry, The Johns Hopkins University School of Medicine, Baltimore, Maryland, USA.; 8 The Scripps Research Institute, La Jolla, California, USA.; 9 Department of Microbiology and Molecular Genetics, University of Pittsburgh, Pittsburgh, Pennsylvania, USA.

**Keywords:** Immunology, Reproductive biology, Antigen, Mouse models, T cells

## Abstract

Pregnancy is an immunological paradox where despite a competent maternal immune system, regulatory mechanisms at the fetoplacental interface and maternal secondary lymphoid tissues (SLTs) circumvent rejection of semi-allogeneic concepti. Small extracellular vesicles (sEVs) are a vehicle for intercellular communication; nevertheless, the role of fetoplacental sEVs in transport of antigens to maternal SLTs has not been conclusively demonstrated. Using mice in which the conceptus generates fluoroprobe-tagged sEVs shed by the plasma membrane or released from the endocytic compartment, we show that fetoplacental sEVs are delivered to immune cells in the maternal spleen. Injection of sEVs from placentas of females impregnated with Act-mOVA B6 males elicited suboptimal activation of OVA-specific CD8^+^ OT-I T cells in virgin females as occurs during pregnancy. Furthermore, when OVA^+^ concepti were deficient in Rab27a, a protein required for sEV secretion, OT-I cell proliferation in the maternal spleen was decreased. Proteomics analysis revealed that mouse trophoblast sEVs were enriched in antiinflammatory and immunosuppressive mediators. Translational relevance was tested in humanized mice injected using sEVs from cultures of human trophoblasts. Our findings show that sEVs deliver fetoplacental antigens to the mother’s SLTs that are recognized by maternal T cells. Alterations of such a mechanism may lead to pregnancy disorders.

## Abstract 2

Extracellular vesicels released by the fetoplacental unit carry paternal antigens to the maternal secondary lymphoid tissues where they are presented to maternal T cells resulting in deficient T cell activation.

## Graphical abstract

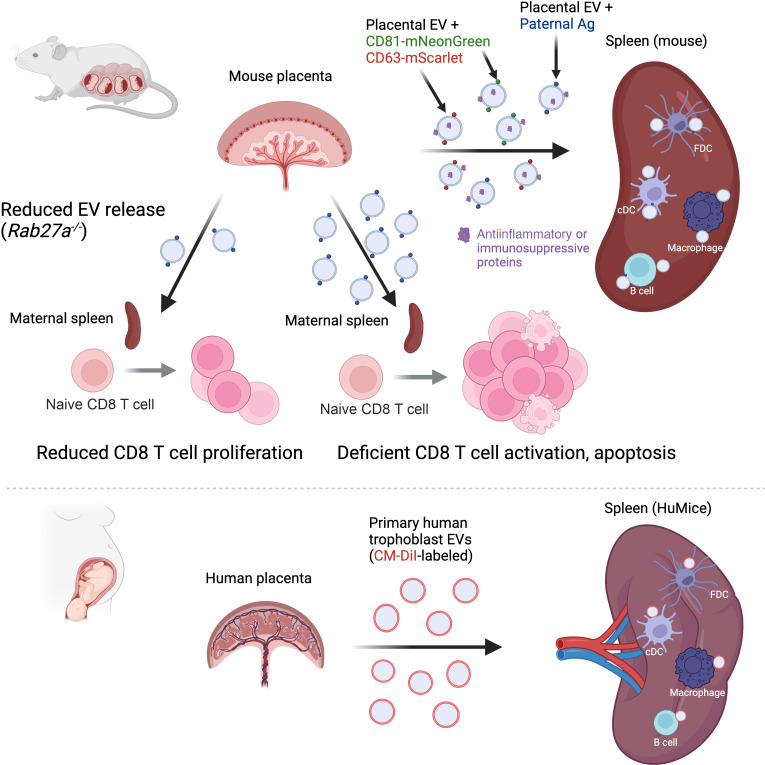

## Introduction

During pregnancy, the fetus and placenta constitute semi-allogeneic components because of the expression of paternal and embryonic antigens (Ags) to which, in most cases, the maternal T and B cells have not been previously exposed or tolerized. Interestingly, a healthy fetoplacental unit is not rejected by the mother’s immune system. This phenomenon occurs even in donor oocytes or surrogate pregnancies, where the conceptus is fully allogeneic. The immunoregulatory mechanisms at the fetomaternal interface and in the maternal lymphoid tissues that prevent immunologic attack on the conceptus, while maintaining competence against pathogens, remain largely unknown.

Like the adaptive immune response elicited by allogeneic transplants, maternal T and B lymphocytes become aware of the presence of non-self-Ags expressed by the conceptus. Studies in mouse models have shown that T cells in the mother’s secondary lymphoid tissues (SLTs) recognize conceptus-derived peptides presented or crosspresented by maternal Ag-presenting cells (APCs) (1, 2). Expansion of maternal CD4^+^ regulatory T cells (Tregs) in combination with clonal deletion or exhaustion of maternal CD8^+^ T cells in the mother’s SLTs is critical to fetomaternal tolerance (1, 3–14). Despite these regulatory mechanisms, a percentage of conceptus-specific maternal CD8^+^ T cells persist as memory T cells after parturition and can differentiate into effector cells after Ag reencounter in a nonpregnancy context (9, 11).

The mechanism(s) by which embryonic and paternal Ags are systemically delivered to maternal immune cells in the mother’s SLTs has not been elucidated. Fetomaternal cell chimerism might be a source of conceptus Ag; nevertheless, fetal cells are infrequent in the maternal blood or lymphoid organs (15, 16). Fetal conventional dendritic cells (cDCs), the equivalents of donor migratory passenger leukocytes in allografts, do not develop until advanced pregnancy (17). Importantly, decidual maternal DCs are not involved in presentation of fetal Ags in the mother’s SLTs, since they remain confined to the pregnant uterus (18). Thus, fetoplacental Ags could reach the mother’s SLTs cell free via extracellular vesicles (EVs) released by the trophoblast and fetus (19).

Although the function of EVs in vivo is largely unknown (20, 21), they represent an efficient platform for Ag delivery from peripheral tissues to SLTs to stimulate allogeneic immune responses following transplantation (22). Passage of small EVs (sEVs; exosomes) constitutes a mechanism of fetal-placental-maternal crosstalk by which proteins, Ags, immunoregulatory mediators, mRNAs, noncoding RNAs, small DNA fragments, metabolites, and lipids are exchanged (19). Trophoblasts shed nuclear aggregates, apoptotic blebs, and different types of EVs into the maternal blood during normal and pathological pregnancies, with increasing levels toward term (23–34). While the cell aggregates are trapped in the pulmonary capillaries, the sEVs circulate systemically and have access to the maternal SLTs. Trophoblast EVs express paternally inherited minor histocompatibility Ags (35), and placental EVs regulate the function of immune and endothelial cells in vitro (36–41). Nevertheless, there is no conclusive evidence that sEVs transport Ag from the fetoplacental unit to the mother’s SLTs, mainly due to the lack of mouse models that allow tracking sEVs in vivo.

In this study, by taking advantage of mouse pregnancy models in which the fetoplacental unit releases fluorochrome-tagged sEVs (e.g., exosomes), we demonstrate that sEVs shed systemically by the fetoplacental unit reach maternal immune cells in the mother’s SLTs. Our models allowed us to distinguish whether the conceptus-derived sEVs captured by maternal immune cells in SLTs were acquired from systemically released vesicles or from sEVs secreted locally by fetoplacental chimeric cells that migrated to the maternal spleen. The conceptus-derived sEVs delivered paternal Ags together with antiinflammatory/immunosuppressive mediators to maternal APCs, which elicited maternal T cell activation with characteristics reminiscent of those previously reported during middle/late gestation in mice (1). Our findings highlight the importance of the physical properties of the sEVs for efficient relay of conceptus Ags to maternal immune cells. Experiments in humanized mice injected with human trophoblast sEVs validated our findings in mice in a relevant translational system.

## Results

### sEVs shed by the fetoplacental unit reach maternal immune cells in SLTs.

**Figure 1:**
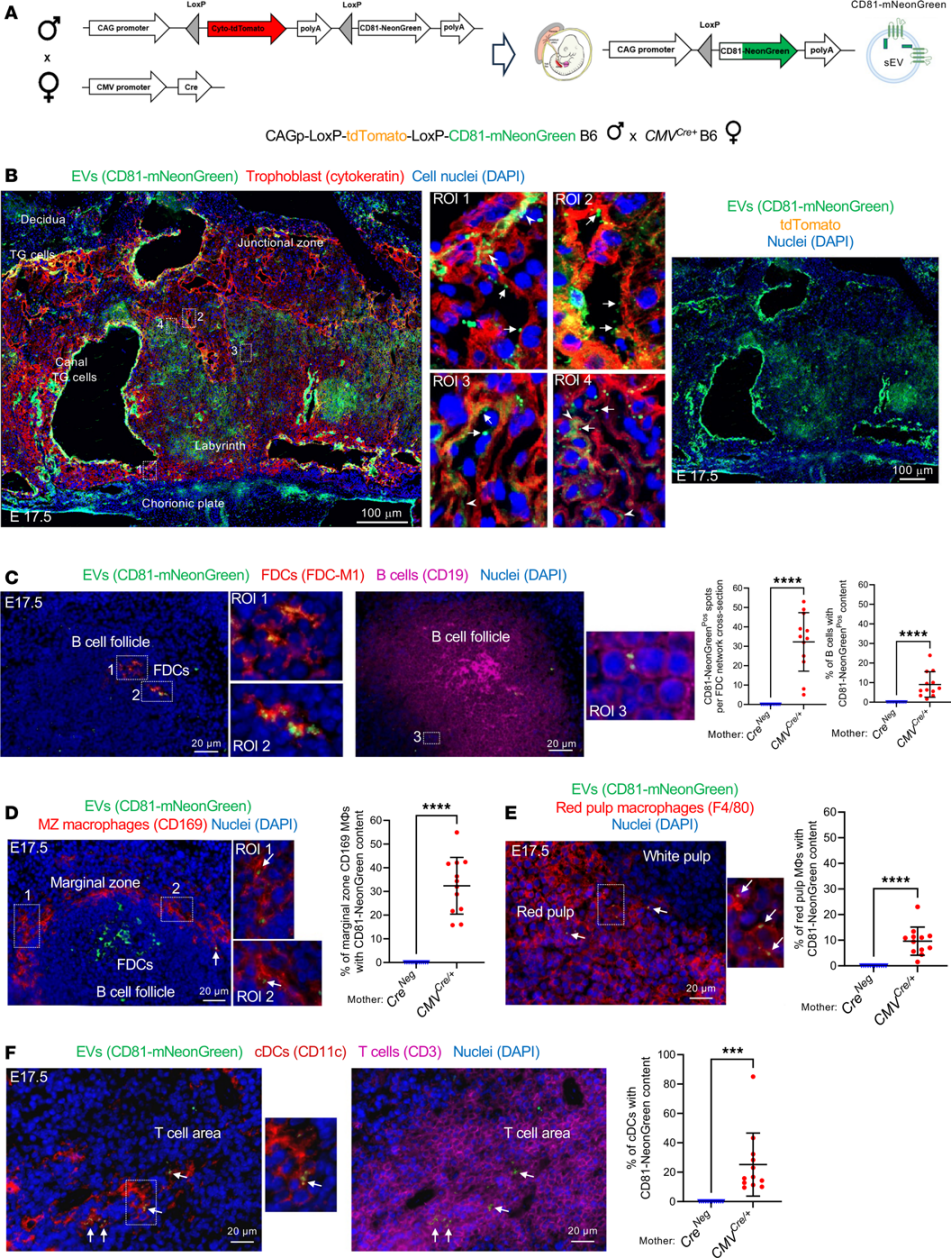
Fetoplacental CD81 sEVs traffic to the maternal spleen. (**A**) Diagram of *tdTomato^LSL^ CD81-mNeonGreen* (Exomap1) B6 male × *CMV^Cre/+^* B6 female pregnancy model. In the presence of Cre, *LoxP* recombination removes cytoplasmic (Cyto) *tdTomato* polyA, leading to downstream transcription of *CD81-mNeonGreen*. CD81-mNeonGreen protein is sorted in fetoplacental sEVs. (**B**) CD81-mNeonGreen on sections of placentas of *CMV^Cre/+^* B6 females impregnated by Exomap1 B6 males. Region of interest (ROI) showing CD81-mNeonGreen^+^ EVs shed from trophoblast cells (arrows) and CD81-mNeonGreen content in trophoblast cell invaginations (arrowheads). Representative of 6–12 placentas from 4 mothers. Original magnification, ×200. TG, trophoblast giant. (**C**–**F**) Detection by microscopy of CD81-mNeonGreen in FDCs (**C**), B cells (**C**), MZ macrophages (**D**), red pulp macrophages (**E**), and cDCs (**F**), in spleens of *CMV^Cre/+^* B6 females impregnated with Exomap1 B6 males and analyzed on E17.5. Original magnification, ×200, ×400. Dot plots in **C**–**F**, quantification on spleen cryosections of FDCs, B cells, MZ macrophages, red pulp macrophages, and cDCs with CD81-mNeonGreen. Images representative of multiple sections from spleens of 4 pregnant females. In **C**–**F**, comparisons by 2-tailed Student’s test. Error bars: means ± SD. ***P < 0.001 and ****P < 0.0001.

We investigated whether sEVs released by the fetoplacental unit reach maternal immune cells in the mother’s SLTs by means of 2 mouse models. In the first model, we used Exomap1 B6 mice encoding a chicken β-actin (CAG) promoter–driven floxed stop cassette containing tdTomato, followed by the sEV-associated marker CD81 fused to mNeonGreen (42). CD81 is normally localized to the plasma membrane, the primary site of shedding of sEVs in most cell types (43). Upon mating homozygous Exomap1 B6 males with homozygous *CMV^Cre/+^* B6 females, the floxed cassette was removed, which was verified by the lack of tdTomato expression in most of the placental cells, resulting in the expression of CD81-mNeonGreen by the trophoblast (Figure 1A). As controls, homozygous Exomap1 B6 males were mated with *Cre^–^* B6 females. Microscopic analysis of placenta cryosections on embryonic day 17.5 (E17.5) revealed CD81-mNeonGreen expression on surface projections of trophoblasts, compatible with shedding of sEVs from the trophoblast plasma membrane to the maternal blood spaces (Figure 1B). The labyrinth trophoblasts contain invaginations of the plasma membrane (44), which explains the detection of CD81-mNeonGreen within internal compartments of trophoblasts (Figure 1B). Expression of CD81-mNeonGreen was particularly strong on canal-associated giant cells lining the vascular canals (Figure 1B). As expected, mNeonGreen was negative and tdTomato was broadly expressed in placentas of *Cre^–^* controls (Supplemental Figure 1; supplemental material available online with this article; https://doi.org/10.1172/jci.insight.186335DS1). In the decidua, CD81-mNeonGreen content was detected in stromal cells, maternal blood endothelial cells, and leukocytes adjacent to the junctional zone (Supplemental Figure 2), which is indicative of uptake of placenta-derived sEVs by maternal decidual cells.

In the maternal spleen on E17.5, CD81-mNeonGreen was found in follicular dendritic cells (FDCs), B cells, marginal zone (MZ) and red pulp macrophages, and cDCs (Figure 1, C–F). CD81-mNeonGreen detected in the maternal spleen was not delivered through shedding of trophoblast fragments, since it did not contain cytokeratin (Supplemental Figure 3A). mNeonGreen was undetectable in placentas or maternal spleen when control *Cre^–^* B6 females were impregnated by Exomap1 B6 males (Supplemental Figure 1 and Supplemental Figure 3B).

**Figure 2:**
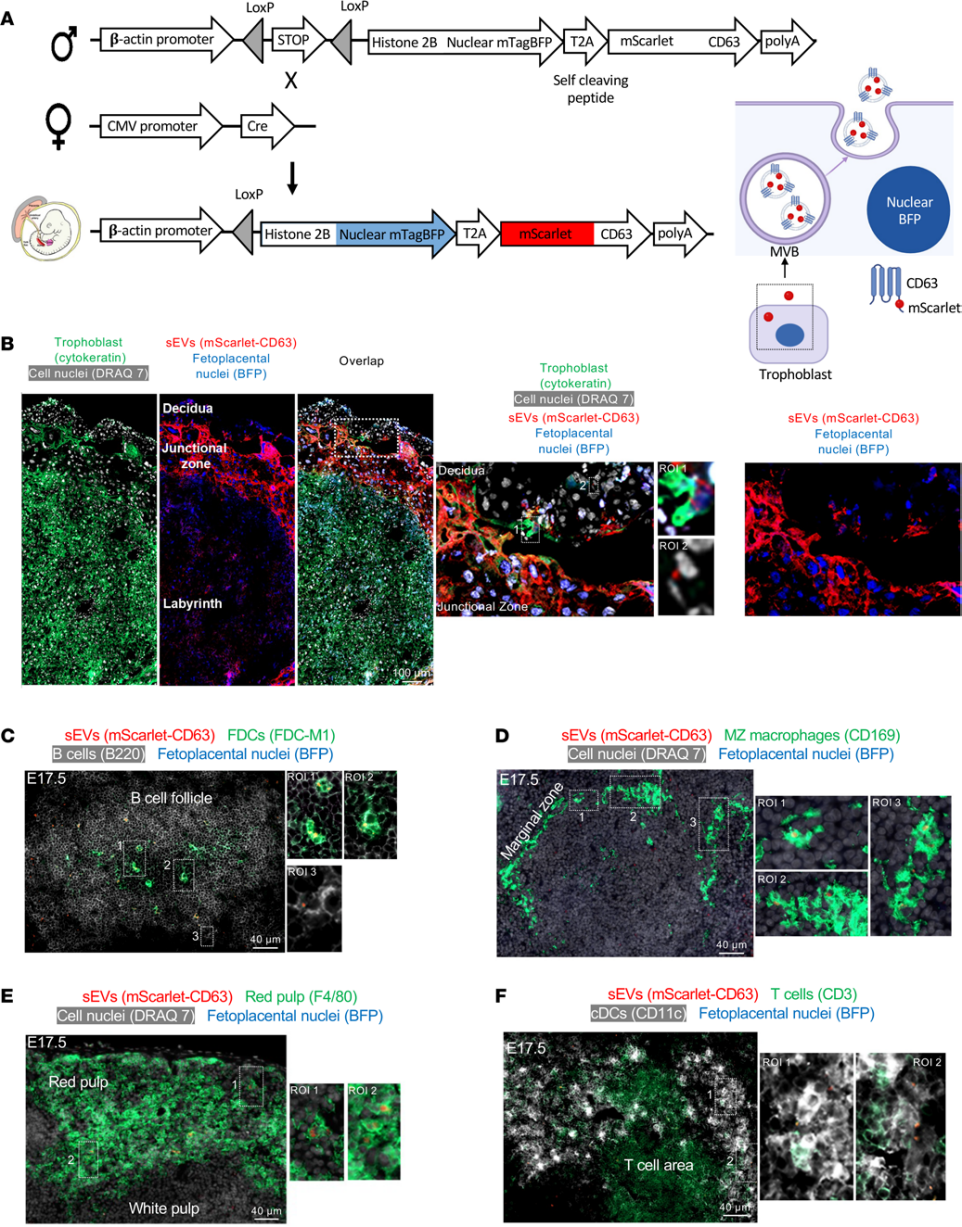
Fetoplacental CD63 sEVs reach the maternal immune cells in the spleen in the absence of detectable fetomaternal chimeric cells. (**A**) Diagram of nuclear-BFP mScarlet-CD63*^LSL^* B6 male × *CMV^Cre/+^* B6 female pregnancy model. Following Cre recombination, fetoplacental cells express nuclear-BFP and generate sEVs bearing mScarlet fused to mouse CD63, both driven by the ubiquitous β-actin promoter. MVB, multivesicular body. (**B**) Detection by microscopy of mScarlet-CD63 sEVs in trophoblast cells identified by their BFP-expressing nuclei, analyzed on E17.5 in *CMV^Cre/+^* B6 females impregnated by nuclear-BFP mScarlet-CD63*^LSL^* B6 males. Inset: interface between the trophoblast and decidua basalis. ROI 1: a trophoblast cell identified by its cytokeratin expression, BFP^+^ nucleus, and content of mScarlet-CD63 sEVs. ROI 2: a maternal cell (BFP^–^ nucleus) with mScarlet-CD63 likely acquired from trophoblast cells invading the decidua. Original magnification, ×200, ×400. Images representative of 4 placentas. (**C**–**F**) Microscopy analysis on E17.5 of cryosections of spleens from *CMV^Cre/+^* B6 females impregnated by nuclear-BFP mScarlet-CD63*^LSL^* B6 males. mScarlet-CD63 is detectable in the maternal FDC network, MZ macrophages, red pulp macrophages, and cDCs. Fetoplacental cells expressing nuclear-BFP were undetectable in the maternal spleen. Images representative of multiple sections of spleens from 4 pregnant females. Original magnification, ×200.

We validated the previous findings in a second mouse in which fetoplacental sEVs were tagged with mScarlet fused to CD63, a molecule highly enriched in sEVs generated in endocytic compartments (43, 45). In addition, to test whether the conceptus-derived sEVs captured by maternal cells in the spleen were acquired from systemically released vesicles, or from sEVs secreted locally by neighboring fetoplacental cells migrated to the maternal spleen, fetoplacental cells releasing mScarlet-CD63–tagged sEVs were engineered to coexpress blue fluorescent protein (BFP) in the nucleus. We generated nuclear-BFP mScarlet-CD63*^LSL^* B6 mice encoding a floxed stop cassette upstream of mTagBFP2 fused to histone 2BC11, followed by mScarlet fused to mouse CD63, driven by the ubiquitous CAG promoter (Figure 2A). The 2 fusion proteins were linked by a self-cleaving peptide. Homozygous nuclear-BFP mScarlet-CD63*^LSL^* B6 mice were mated with homozygous *CMV^Cre/+^* B6 females, and the presence of fetoplacental mScarlet-CD63 content and of cells with BFP-labeled nuclei were analyzed in the placenta and maternal spleen on E17.5. Unlike the plasma membrane–resident CD81, most CD63 is rapidly endocytosed from the cell surface to endosomes/multivesicular bodies, inside which sEVs also originate (43, 45). It has been widely assumed that most CD63-bearing sEVs are derived from intraluminal vesicles generated in the endocytic compartment by reverse budding of the limiting membrane of multivesicular bodies. However, recent evidence indicates that CD63 endocytosis drastically reduces its vesicular secretion from the cell and that most of the CD63-carrying sEVs are shed directly from the plasma membrane (46). This phenomenon could be accentuated in our mScarlet-CD63 transgenic model, since overexpression of CD63 drives its plasma membrane accumulation due to competition for limited quantities of clathrin adaptor complex AP-2 that is required for CD63 endocytosis (46).

Analysis of placenta cryosections showed mScarlet-CD63 in a punctate pattern in trophoblast cells with BFP-labeled nuclei, in the junctional zone and the labyrinth (Figure 2B). As expected, decidual maternal cells did not express nuclear-BFP, except for invading trophoblast cells with mScarlet-CD63 content (Figure 2B). Occasionally, we detected maternal decidual cells (BFP^–^) with mScarlet-CD63 content, indicative of transfer of sEVs from the conceptus to the decidual maternal cells (Figure 2B). In the mother’s spleen, mScarlet-CD63 was found in FDCs, B cells, MZ and red pulp macrophages, and cDCs (Figure 2, C–F). Conceptus-derived cells with BFP-labeled nucleus and mScarlet-CD63 content were undetectable, indicative of minimal or null contribution of fetoplacental chimeric cells in the passage of conceptus-derived sEVs in the mother’s spleen (Figure 2, C–F). As expected, mScarlet-CD63 and BFP were negative in placentas and maternal spleens of control *Cre^–^* B6 females mated with nuclear-BFP mScarlet-CD63*^LSL^* B6 males. In summary, sEVs released systemically by the fetoplacental unit reach maternal immune cells in the spleen.

### Fetoplacental sEVs deliver paternal Ags to maternal immune cells in the spleen.

**Figure 3:**
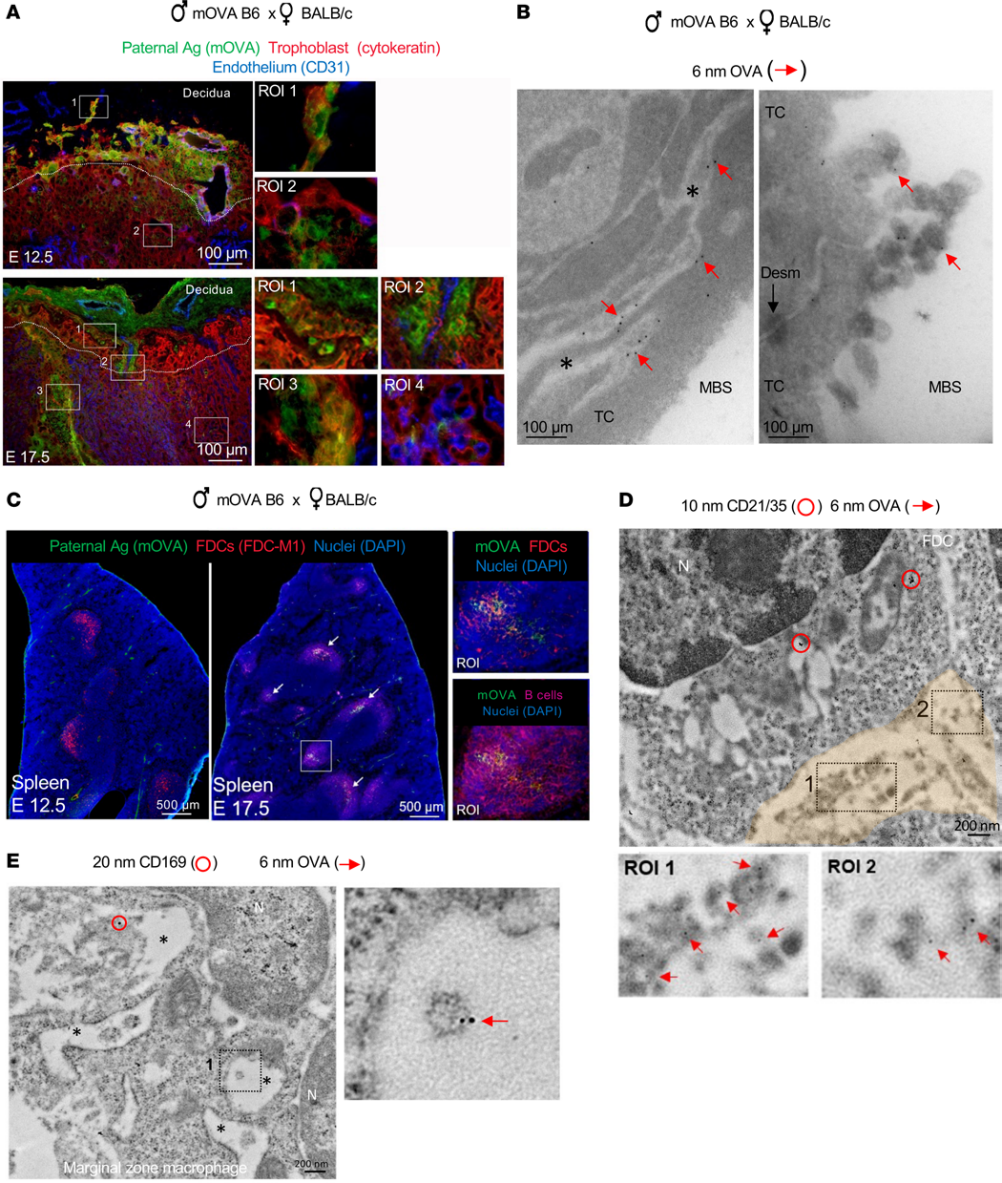
Fetoplacental Ags are relayed to maternal immune cells via sEVs. (**A**) Microscopy of expression of mOVA on cryosections of placentas of BALB/c females impregnated with mOVA B6 males. Dotted line: limit between the junctional zone and the labyrinth. Representative of 6–8 placentas from 3 pregnant females. Original magnification, ×200. (**B**) Detection of mOVA by IEM on sections of placentas of BALB/c females mated with mOVA B6 males. Asterisk, cell membrane invaginations in trophoblast cells. Original magnification, ×20,000, ×80,000. TC, trophoblast cell; MBS, maternal blood space; Desm, desmosome. (**C**) Detection by microscopy of mOVA on cryosections of spleens of BALB/c females impregnated with heterozygous mOVA B6 males. Representative of spleens from 4 pregnant mice per time point. Original magnification, ×200. (**D**) IEM of OVA Ag (6 nm gold) on sEVs captured by an FDC identified by expression of CD21/35 (10 nm gold, red circles) in the spleen of a BALB/c female mated with an mOVA B6 male and analyzed on E17.5. Fetoplacental sEVs expressing OVA in the endocytic compartment (pseudocolored) of the FDC. ROIs: detail of OVA Ag (6 nm gold, arrows) on fetoplacental sEVs internalized by the FDC. Original magnification, ×20,000, ×80,000. N, nucleus. (**E**) IEM of fetoplacental sEVs bearing paternal mOVA (6 nm gold, arrow) within interconnected vesicles of MZ CD169^+^ macrophages (20 nm gold, red circle) in the spleen of a BALB/c female mated with a mOVA B6 male and analyzed on E17.5. Original magnification, ×20,000, ×80,000.

Next, we investigated if the fetoplacental sEVs captured by maternal immune cells in the maternal spleen transport paternal Ags. We used a well-established semi-allogeneic pregnancy model in which WT BALB/c (H2^d^) females were impregnated with B6 (H2^b^) mice hemizygous for the membrane-bound chicken ovalbumin (Act-mOVA) transgene (mOVA B6). In this model, mOVA functions as a paternal surrogate Ag expressed by the junctional zone cells, trophoblast giant cells at the interface between the junctional zone and decidua, and canal giant trophoblast and labyrinth trophoblasts (Figure 3A). By immuno-electron microscopy (IEM), mOVA was detected on the surface and plasma membrane invaginations of labyrinth trophoblast cells and on sEVs shed from trophoblast cells to the maternal blood spaces (Figure 3B).

In the maternal SLTs, mOVA Ag was detected mostly on maternal FDCs in the spleen, as previously reported in a different female strain combination (Figure 3C) (1, 47). In the spleen, the amount of mOVA Ag detected in FDCs increased from E12.5 to E17.5 (Figure 3C). Next, by IEM on ultra cryosections of the mother’s spleen, we investigated the format by which the conceptus-derived mOVA Ag reaches the maternal FDCs and whether mOVA was present in other subsets of maternal immune cells at levels we found undetectable by fluorescence microcopy. Ultrastructural analysis revealed that FDCs retained conceptus-derived mOVA on sEVs internalized in clusters inside glomerulus-like vesicular compartments containing complex membrane labyrinthine infoldings, an ultrastructural feature of FDCs (Figure 3D and Supplemental Figure 4A). sEVs bearing mOVA Ag were also detected within interconnected endocytic vesicles that run across MZ CD169 macrophages (Figure 3E and Supplemental Figure 4B) or next to CD11c cDCs (Supplemental Figure 4C). In summary, at least a fraction of fetoplacental Ags are delivered to immune cells in the maternal spleen via sEVs.

### EVs carrying paternal Ags in peripheral blood of pregnant mice.

**Figure 4:**
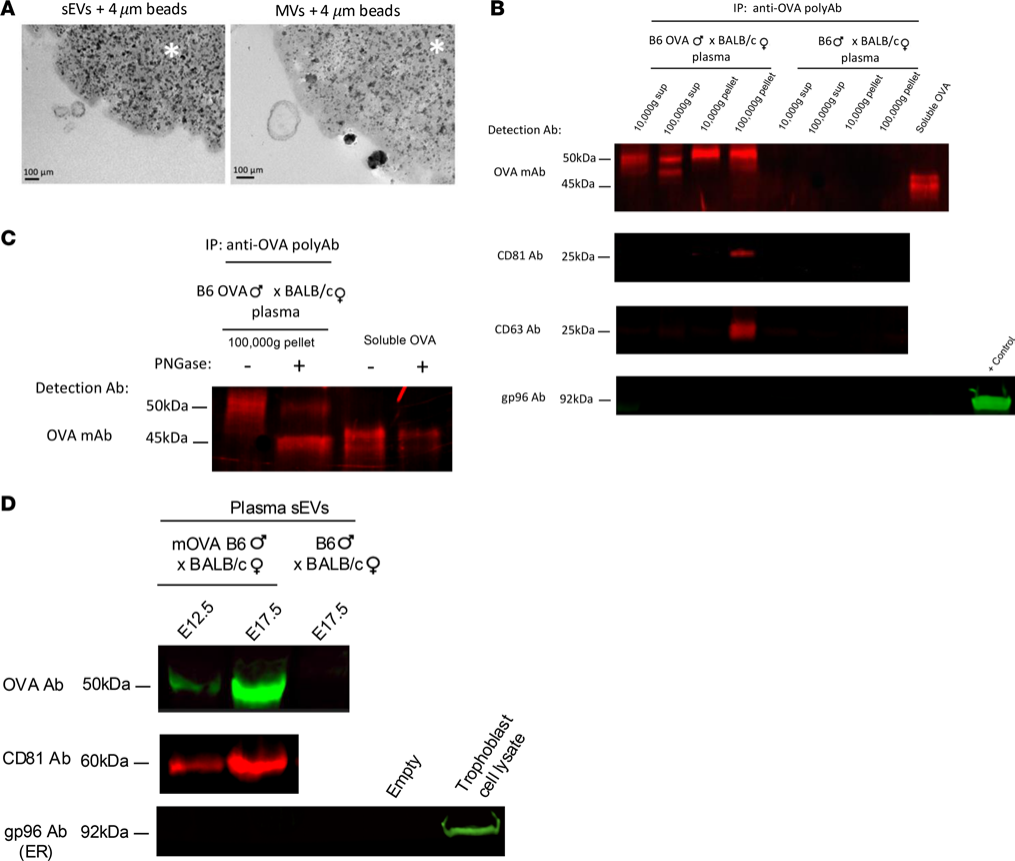
Maternal plasma carries paternal Ag associated with EVs and in soluble form. (**A**) Electron microscopy of pregnant mouse plasma sEVs and MVs bound to the OVA Ab–coated beads used for immunoprecipitation. Original magnification, ×40,000. (**B**) Immunoprecipitation and Western blot detection of OVA, the sEV-associated markers CD81 and CD63, and the ER marker gp96 in MVs and sEVs purified from plasma of BALB/c females impregnated with mOVA B6 males or control WT B6 males (E12.5–E17.5) and in 10,000*g* and 100,000*g* EV-cleared plasma from the same females. (**C**) PNGase digestion of the immunoprecipitate restores the OVA MW to the size of the positive control, which indicates that the OVA released by the conceptus is N*-*glycosylated. (**D**) Analysis by Western blot of paternal Ag mOVA on sEVs purified on E12.5 and E17.5 from pooled plasma of BALB/c females impregnated by mOVA B6 or control B6 males. CD81 and gp96 were included as EV-associated and EV exclusion markers, respectively. In **B** and **C** results are representative of 3 different samples.

The finding of sEVs bearing the paternal Ag mOVA in the maternal spleen was indicative of the spread of conceptus mOVA via maternal blood, because the spleen lacks afferent lymphatics. To validate this, we isolated fetoplacental EVs carrying mOVA from the maternal plasma of WT BALB/c females impregnated with mOVA B6 or control WT B6 males (E17.5). Maternal plasma was centrifuged successively at 10,000*g* and 100,000*g* to pellet microvesicles (MVs) and sEVs, respectively. The EV fractions and the corresponding EV-cleared supernatants were immunoprecipitated with magnetic beads coated with a polyclonal rabbit anti-OVA Ab (Figure 4A). Western blot analysis of the immunoprecipitated material using a mouse monoclonal Ab against OVA demonstrated the presence of OVA in the trophoblast-derived MVs and sEVs and as soluble Ag in the EV-free fraction (Figure 4B). As a control, OVA was undetectable in immunoprecipitates of EVs and EV supernatants from plasma of WT BALB/c females impregnated by WT B6 males (Figure 4B). The purity of the sEVs was verified by their positivity for the sEV-associated markers CD81 and CD63 and absence of the ER-associated marker gp96 (Figure 4B). Treatment of the samples with Peptide:N-glycosidase F (PNGase) before analysis indicated that the higher MW of the OVA in the plasma EVs and its supernatants compared with control soluble OVA was due to N-linked glycosylation (Figure 4C). Next, we investigated if the load of fetoplacental sEVs carrying mOVA in maternal blood varies during gestation. Western blot analysis of fetoplacental sEVs isolated by microfluidic tangential flow filtration on E12.5 and E17.5 from pooled plasma (3 mice per variable) of BALB/c females impregnated by mOVA B6 or control B6 males showed that the amount of mOVA carried on trophoblast sEVs in the maternal circulation increased substantially from E12.5 to E17.5 (Figure 4D), which is consistent with the amount of OVA detected in the maternal spleen at the same time points (Figure 3C). To rule out the number of mOVA^+^ fetoplacental units per mouse as a confounding variable, the pregnant mice from which the plasmas were pooled had the same number of concepti. Thus, the surrogate paternal Ag mOVA is shed to the maternal blood through sEVs and in soluble form, and the amount of mOVA released via sEVs to the maternal circulation is augmented during gestation.

### Trophoblasts release EVs carrying paternal Ags.

**Figure 5:**
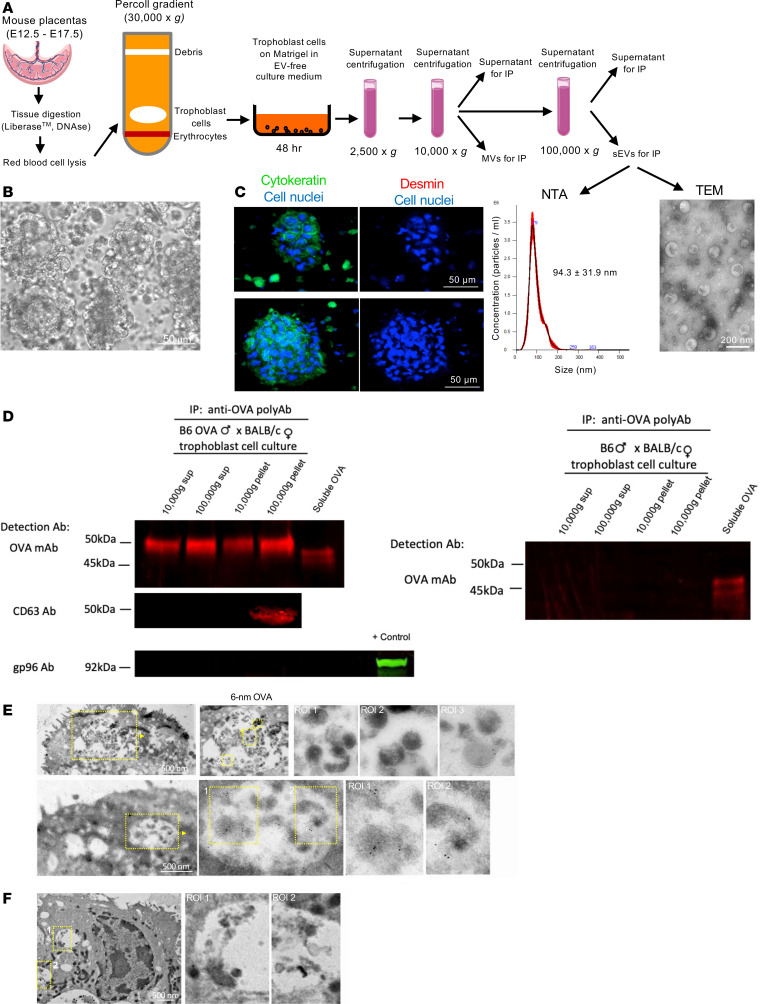
sEVs released by mouse primary trophoblast cultures bear paternal Ag. (**A**) Methodology used to generate primary cultures of mouse trophoblast cells and purify EVs from the culture supernatants. NTA, nanoparticle tracking analysis; TEM, transmission electron microscopy. (**B**) Bright-field microscopy showing clusters of mouse trophoblast cells after 48 hours of culture. (**C**) Microscopy of cytospins of primary cultures of mouse trophoblast cells showing cytokeratin^+^ trophoblast cell clusters with minimal contamination with desmin^+^ decidual cells. (**D**) Immunoprecipitation and Western blot of purified EVs and EV-clarified supernatants from mouse primary trophoblast cultures from placentas of BALB/c females impregnated by mOVA B6 (on the left) or control WT B6 males (on the right). Samples were immunoprecipitated with a polyclonal Ab against OVA and analyzed by Western blot for detection of OVA with a monoclonal Ab, the sEV-associated marker CD63, and the ER marker gp96. Results representative of 2 independent experiments. (**E**) IEM of primary cultures of trophoblast cells from placentas (E12.5–E17.5) of BALB/c females impregnated with mOVA B6 males. Images show multivesicular bodies with intraluminal vesicles expressing OVA labeled with 6 nm gold. (**F**) IEM of primary cultures of trophoblast cells from placentas of BALB/c females impregnated with control WT B6 males (E12.5–E17.5) depicting multivesicular bodies with intraluminal vesicles on which OVA was not detected. In **E** and **F**, images are representative of 3 samples. Original magnification, ×40,000.

The EVs carrying mOVA isolated from peripheral blood of pregnant mice could have been released by the placenta, the fetus, or both. Therefore, we analyzed whether the mouse trophoblast releases EVs carrying the paternal Ag mOVA using primary trophoblast cell cultures from placentas (E12.5–E17.5) of WT BALB/c females impregnated by mOVA B6 or control WT B6 males (Figure 5A). The mouse primary trophoblast cell cultures were composed predominantly of pan-cytokeratin^+^ trophoblast cells, mostly organized in clusters, with minimal contamination of desmin^+^ decidual cells (Figure 5, B and C). EVs were isolated from 48-hour culture supernatants by sequential ultracentrifugation (Figure 5A). Analysis by electron microscopy and nanoparticle tracking analysis verified the purified samples were composed of a homogeneous population of sEVs (Figure 5A).

By Western blot, and consistent with the findings in plasma EVs from pregnant mice, mOVA was detected in 48-hour trophoblast culture supernatant-derived MVs and in CD63^+^ sEVs and respective EV-free centrifuged fractions. mOVA was absent in their counterparts isolated from trophoblast culture supernatant generated from placentas of pregnant BALB/c females mated with control WT B6 males (Figure 5D). IEM of primary mouse trophoblast cell cultures verified the presence of mOVA on intraluminal vesicles inside multivesicular bodies and on the surface of trophoblast cells generated from placentas of WT BALB/c females impregnated by mOVA B6 males (Figure 5E and Supplemental Figure 5A). The release of sEVs bearing mOVA by trophoblast cells into the extracellular space was also verified by IEM (Supplemental Figure 5B). No labeling with 6 nm gold OVA Ab was found on control trophoblast cultures from placentas of BALB/c female mated with WT B6 males (Figure 5F).

**Figure 6:**
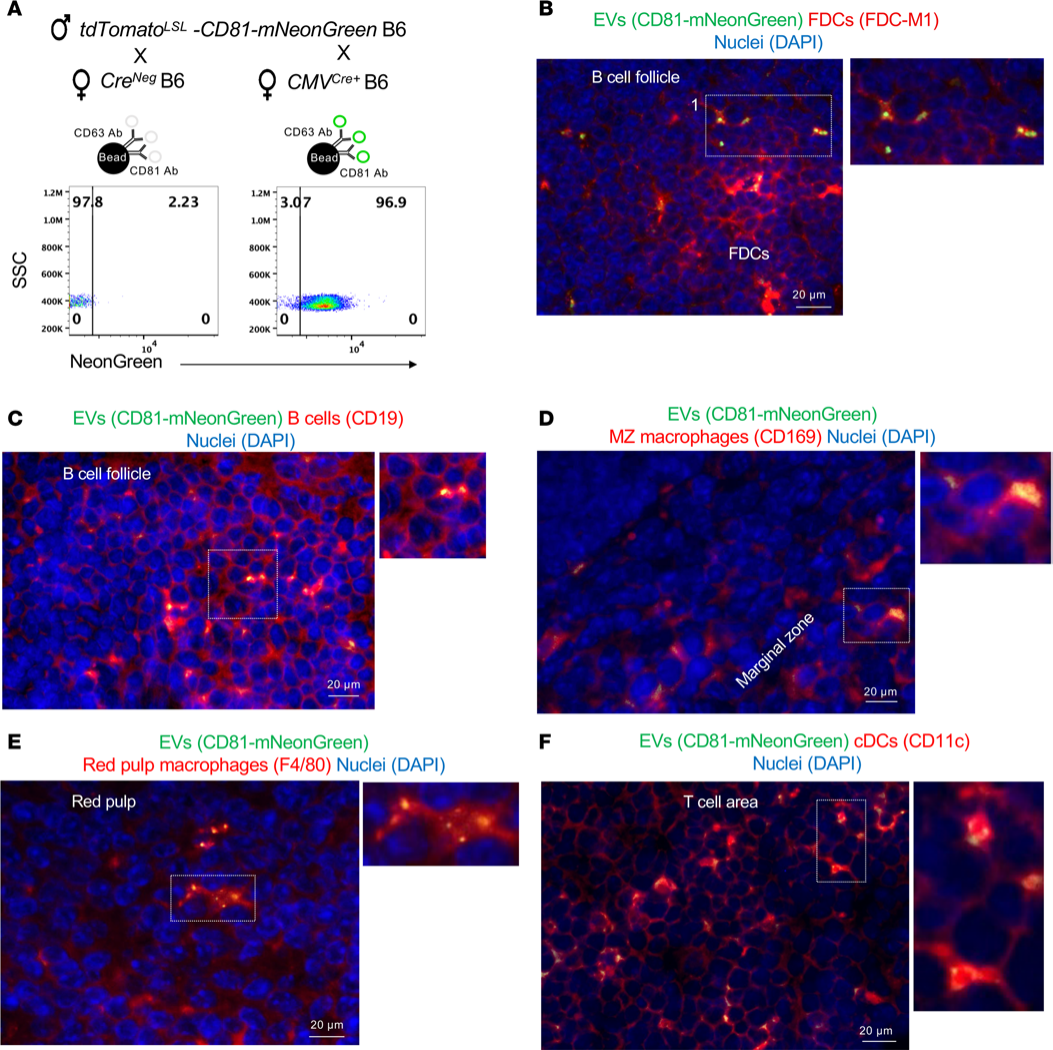
An i.v. injection of sEVs from cultures of trophoblasts mimics the traffic of endogenous fetoplacental sEVs in the mother’s spleen. (**A**) Flow cytometry analysis of EV-bead complexes containing sEVs isolated from primary cultures of trophoblasts from placentas (E14.5–E17.5) of *CMV^Cre+^* B6 females (or control *Cre^–^* B6 females), both impregnated with tdTomato*^LSL^* mNeonGreen-CD81 (Exomap1) B6 males. The sEVs were captured by beads coated with CD63 and CD81 Ab. SSC, side scatter. (**B**–**F**) Detection of sEVs purified from cultures of trophoblasts from placentas (E14.5–E17.5) of *CMV^Cre+^* B6 females × Exomap1 B6 males injected i.v. in B6 virgin females, on cryosections of spleens labeled for identification of FDCs (**B**), B cells (**C**), MZ macrophages (**D**), red pulp macrophages (**E**), or cDCs (**F**). Images are representative of 4 mice. Original magnification, ×200.

Importantly, sEVs isolated from trophoblast cultures of placentas (E12.5–E17.5) from *CMV^Cre/+^* B6 females impregnated with CAGp-tdTomato*^LSL^*-mNeonGreen-CD81 B6 (Exomap1) males expressed mNeonGreen (Figure 6A) and, when i.v. injected into virgin B6 females, recapitulated the trafficking of fetoplacental sEVs to the mother’s immune cells in the spleen detected during gestation (Figure 6, B–F).

### sEVs are an efficient vehicle to deliver conceptus Ags to the maternal spleen.

**Figure 7:**
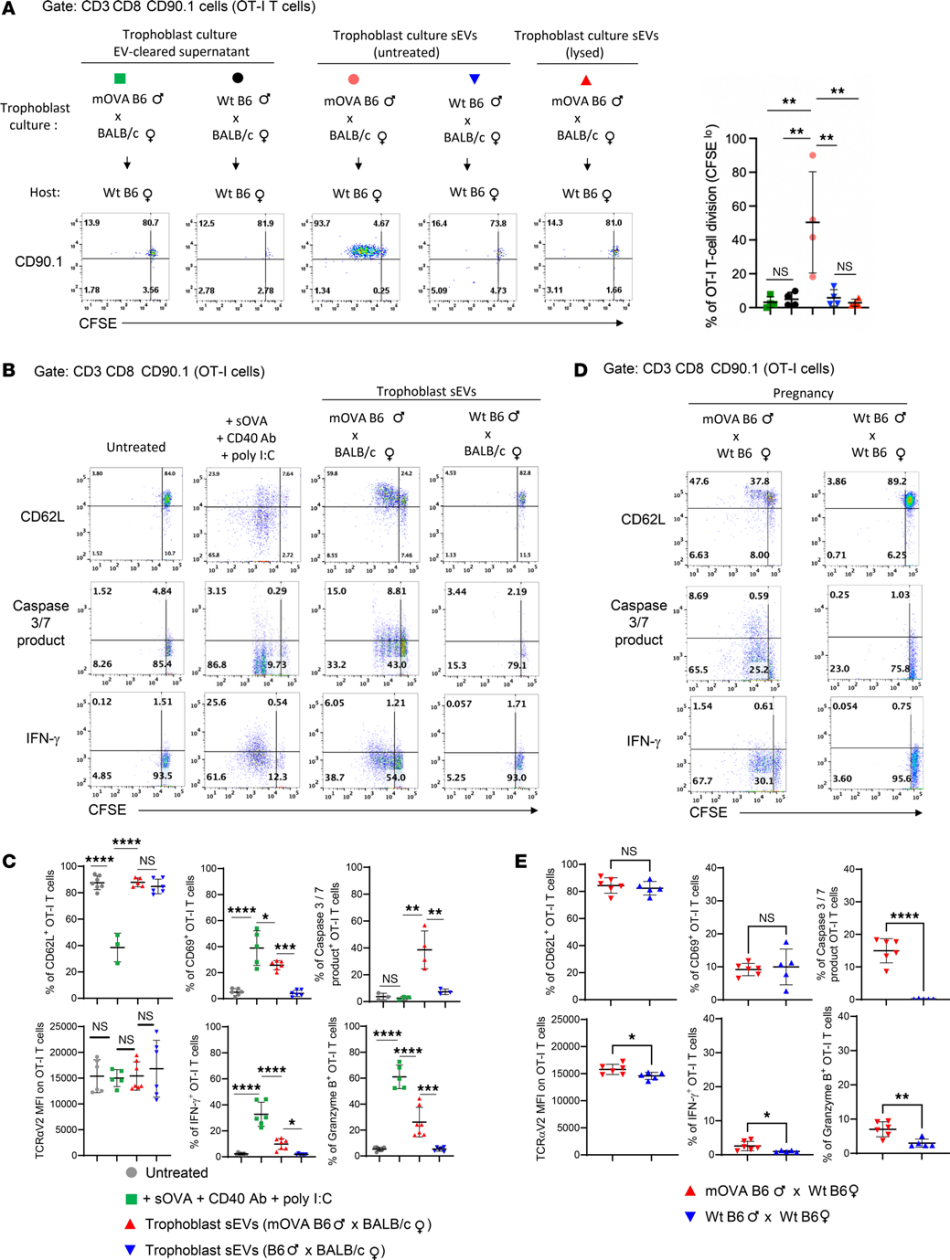
T cells in the maternal spleen recognize paternal Ag on trophoblast sEVs. (**A**) Flow cytometry of proliferation of i.v. injected, CFSE-labeled OT-I CD8 T cells (CD90.1) in spleens of WT virgin female B6 mice (CD90.2) i.v. injected 24 hours later with EV-cleared supernatants or intact or lysed sEVs from cultures of trophoblast cells from BALB/c females impregnated with mOVA B6 or control WT B6 males. On the dot plot on the right, each dot represents 1 mouse. (**B**) Representative flow cytometry of splenocytes of B6 virgin females (CD90.2) i.v. injected with CFSE-labeled OT-I CD8^+^ T cells (CD90.1) and treated i.v. 24 hours later with sEVs from primary trophoblast culture supernatants from BALB/c females impregnated with mOVA B6 males or control WT B6 males. As a positive control of OT-I cell activation, a group was injected i.p. with soluble OVA + agonistic CD40 Ab + poly I:C, 24 hours after the OT-I cell transfer. Experiments were analyzed 2 days after sEV injection. (**C**) Quantification of the results shown in **B**; each dot represents 1 mouse. (**D**) FACS analysis of i.v. administered OT-I T cells (CD90.1) in spleens of females (CD90.2) mated with mOVA or WT B6 male mice. OT-I T cells were i.v. infused on E13.5, and splenocytes were FACS-analyzed 2 days later. (**E**) Quantification of the results in **D**; each dot represents 1 mouse. In **A** and **C**, comparisons by 1-way ANOVA with multiple comparisons. In **E**, comparisons by 2-tailed Student’s test. Error bars: means ± SD. *P < 0.05, **P < 0.01, ***P < 0.001, ****P < 0.001.

Because mouse trophoblasts release the surrogate paternal Ag mOVA via EVs and in a vesicle-free format(s), we compared the efficiency by which trophoblast sEVs deliver immunoreactive mOVA Ag to the spleen with that of soluble, non-EV-associated mOVA shed by the trophoblasts and collected from EV-cleared trophoblast culture supernatants. We tested this in B6 virgin female mice (CD90.2 congenic) i.v. injected with CFSE-labeled TCR transgenic CD8^+^ OT-I T cells, the latter CD90.1 congenic and specific against the OVA-peptide SIINFEKL presented by the B6 MHC class I molecule H2-K^b^. An i.v. administration of sEVs isolated from primary cultures of mOVA B6 male × WT BALB/c female trophoblasts triggered, in the mouse spleens, higher proliferation of OT-I cells than that elicited by an equivalent load (based on Western blot) of mOVA Ag contained in concentrated 100,000*g* supernatants and passed through a 20 nm filter (EV-clear supernatants), collected from the same primary cultures of trophoblast (Figure 7A). As controls, i.v. injection of sEVs or EV-cleared supernatants from primary cultures of WT B6 male × WT BALB/c female trophoblasts elicited minimal OT-I cell division (Figure 7A). The capacity of the sEVs from cultures of mOVA B6 male × WT BALB/c female trophoblasts decreased substantially when the vesicles were disrupted and dialyzed before i.v. injection (Figure 7A). The dialysis of the sEVs was done to retain the mOVA molecules in the lysate injected and, importantly, to remove the detergent used to break down the vesicles. Thus, trophoblast sEVs are a cell-free platform to deliver Ag to the maternal SLTs, and their efficiency to relay Ag to the mother’s immune cells depends on the physical characteristics of the vesicles (intact vesicles, ~100 nm in size).

### Trophoblast sEVs promote suboptimal activation of maternal T cells in the spleen.

Next, we tested if blood-borne trophoblast sEVs carrying mOVA promote canonical activation, differentiation, cell death, or exhaustion of OT-I CD8^+^ T cells. WT B6 virgin female mice (CD90.2) i.v. injected with CFSE-labeled OT-I cells (CD90.1 congenic) were treated i.v. with a single bolus injection of sEVs purified from primary cultures of mOVA B6 male × WT BALB/c female trophoblasts or purified from control WT B6 male × WT BALB/c female trophoblast cultures. The OT-I cell response in the spleen was analyzed by flow cytometry 2 days after sEV injection. Administration of trophoblast sEVs carrying mOVA promoted OT-I cell proliferation but with significantly less downregulation of CD62L and reduced expression of the early activation marker CD69, when compared with controls in which optimal OT-I cell activation was induced by injection of soluble OVA + poly I:C + agonistic CD40 Ab (Figure 7, B and C). The percentage of splenic OT-I cells undergoing apoptosis, based on generation of a fluorogenic product of activated caspase-3, increased in response to administration of trophoblast sEVs bearing mOVA, compared with positive controls (Figure 7, B and C). We detected no difference of expression of the TCRαV2 on the OT-I cells between groups (Figure 7C). Intracellular IFN-γ and granzyme B were barely detectable in OT-I cells that proliferated in response to trophoblast sEVs carrying mOVA (Figure 7, B and C), which suggested decreased differentiation into effector CD8^+^ T cells. Injection of trophoblast sEVs bearing mOVA did not significantly upregulate expression of the exhausted T cell–associated markers TIM-3, Lag3, and PD-1 on proliferating OT-I cells, as compared with OT-I cells of controls injected with soluble OVA + poly I:C + agonistic CD40 Ab (Supplemental Figure 6). OT-I cells did not proliferate in control mice that did not receive trophoblast sEVs or that were injected with sEVs isolated from control WT B6 male × WT BALB/c female trophoblast cultures (Figure 7, B and C). A similar response was detected in the mother’s spleen in OT-I cells i.v. transferred between E13.5 and E15.5 (Figure 7, D and E). Thus, in our model, delivery of a surrogate paternal Ag to the spleen through blood-borne trophoblast sEVs elicits proliferation of OT-I cells with increased percentage of apoptotic cells and decreased expression of effector T cell markers compared with the controls.

### Trophoblast sEVs are enriched in immunoregulatory proteins.

**Figure 8:**
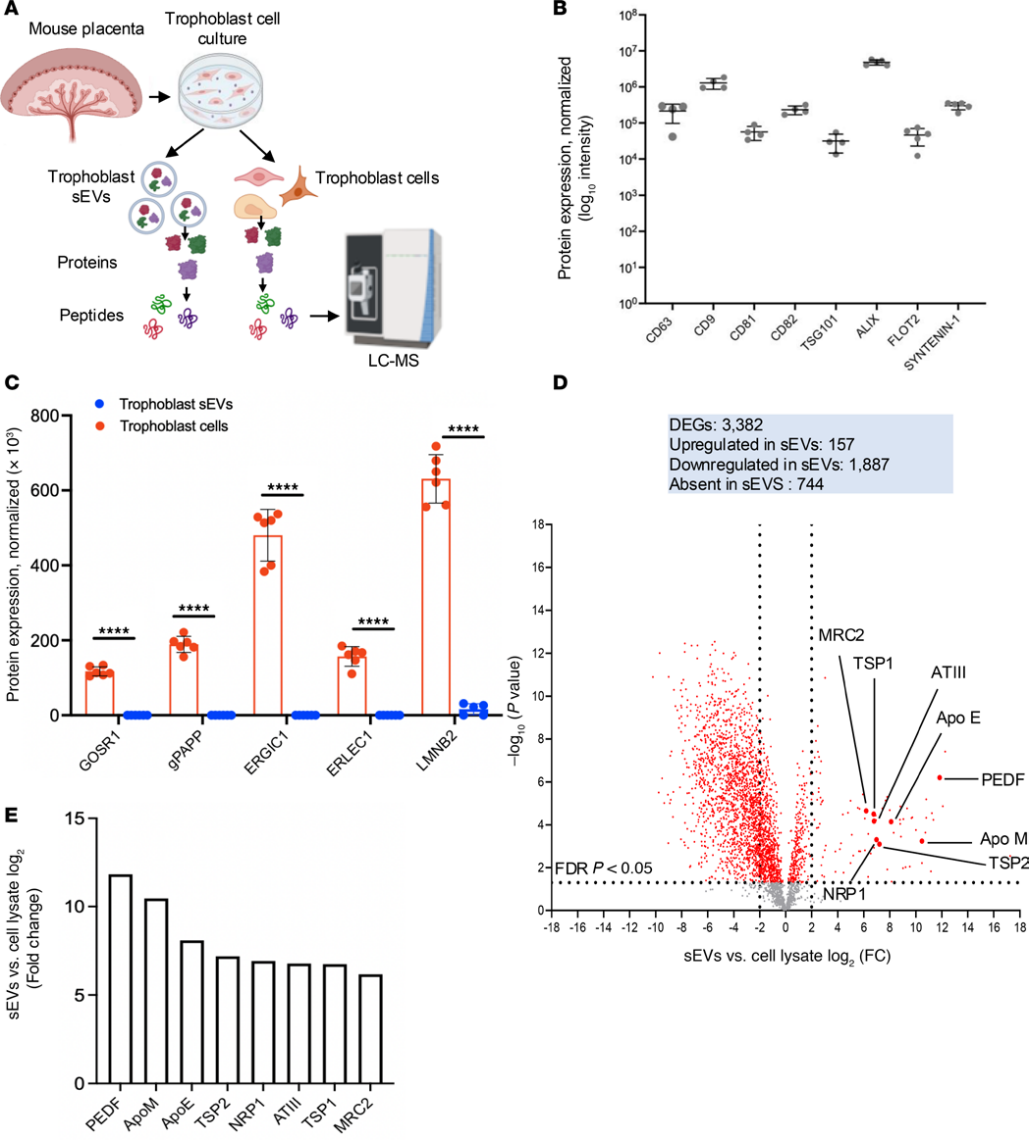
Characterization by high-resolution LC-MS of protein cargo in mouse trophoblast sEVs and the parent trophoblast cells. (**A**) Approach used for comparative analysis by high-resolution LC-MS of the proteome of mouse trophoblast sEVs and their parent cells. (**B**) Normalized expression of sEV biomarkers in trophoblast sEV samples analyzed by LC-MS. Each dot represents an independent trophoblast sEV sample. (**C**) Comparative quantitative expression of sEV exclusion proteins in trophoblast cells versus trophoblast sEVs. Each dot corresponds to 1 independent trophoblast sEV or trophoblast cell sample. GOSR1, Golgi SNAP receptor complex member 1; gPAPP, Golgi-resident PAP-specific 3′-phosphatase-coupled sulfotransferase; ERGIC1, endoplasmic reticulum-Golgi intermediate compartment 1 protein; ERLEC1, endoplasmic reticulum lectin 1; LMNB2, Lamin B2. (**D**) Volcano plot obtained by quantitative analysis by LC-MS of the proteome of trophoblast sEVs versus trophoblast cells. The horizontal dotted line indicates the FDR cutoff line set at *P* < 0.05. The vertical dotted lines represent 2-fold change cutoff. DEGs, differentially expressed genes; FC, fold-change. (**E**) FC increase of individual proteins in trophoblast sEVs versus trophoblast cells indicated in the volcano plot in **E**. (**A**) Created in BioRender. Powell, J. 2025. https://BioRender.com/go3g2fu ****P < 0.0001.

In mice, delivery of fetoplacental Ags to maternal SLTs has been shown to lead to deficient activation, deletion, or exhaustion of T cells and generation of FoxP3^+^ Tregs (1, 3–14). Here, we demonstrated that trophoblast sEVs deliver paternal Ags to the maternal spleen, a phenomenon associated with suboptimal activation of maternal CD8^+^ T cells. Thus, we investigated if trophoblast sEVs, aside from bearing paternal Ags, also carry immunoregulatory proteins that may restrain the immunogenic function of maternal APCs in SLTs. To test this, the proteome of 6 independent samples of sEVs purified by microfluidic tangential filtration from supernatants of mouse primary trophoblast cell cultures was analyzed by high-resolution liquid chromatography and mass spectrometry (LC-MS) and compared with the proteome of the parent trophoblast cells (Figure 8A). The trophoblast sEV samples were highly pure based on their relatively high content of the EV-associated markers CD63, CD9, CD81, CD82, TSG101, ALIX, flotillin 2, and syntenin-1 (Figure 8B), as well as proteins enriched in EVs, the latter including subunits of the ESCRT machinery, members of the Rab family, SNAREs and heat shock proteins, and other chaperones (Supplemental Table 1). Importantly, the purified trophoblast sEVs lacked or contained minimal amounts of proteins of the Golgi apparatus, ER, or nuclear membrane, all considered EV exclusion markers (Figure 8C) (48). Quantitative analysis revealed that of a total of 3,382 proteins detected by LC-MS, 157 were upregulated, 1,887 were downregulated, and 744 were absent in trophoblast sEVs compared with those found in the parent cell extracts (Figure 8D). The content of several proteins with recognized antiinflammatory or immunosuppressive effects was substantially enriched in trophoblast sEVs as compared with parent cells (Figure 8, D and E, and Supplemental Table 2). These included pigment epithelium-derived factor that prevents DC maturation and promotes Tregs’ function and neuropilin-1 that maintains Tregs and sensitizes CD4^+^ T cells to the inhibitory function of semaphorin 3A at the APC–T cell synapse (Figure 8, D and E, and Supplemental Table 2). In comparison with trophoblast cells, trophoblast sEVs carried considerably higher content of apolipoproteins M and E, thrombospondin 1 and 2, antithrombin III, and mannose receptor 2, which, through different pathways, prevent NF-κB activation and the subsequent secretion of the pro-inflammatory cytokines IL-1β, IL-6, and TNF-α, while promoting secretion of IL-10 by DCs and Tregs (Figure 8, D and E, and Supplemental Table 2). Thus, trophoblast sEVs carry a cargo of antiinflammatory and immunosuppressive proteins that are selectively enriched in the vesicles as compared with the parent trophoblast cells.

### Effect of release of conceptus sEVs on recognition of paternal Ag by maternal T cells.

**Figure 9:**
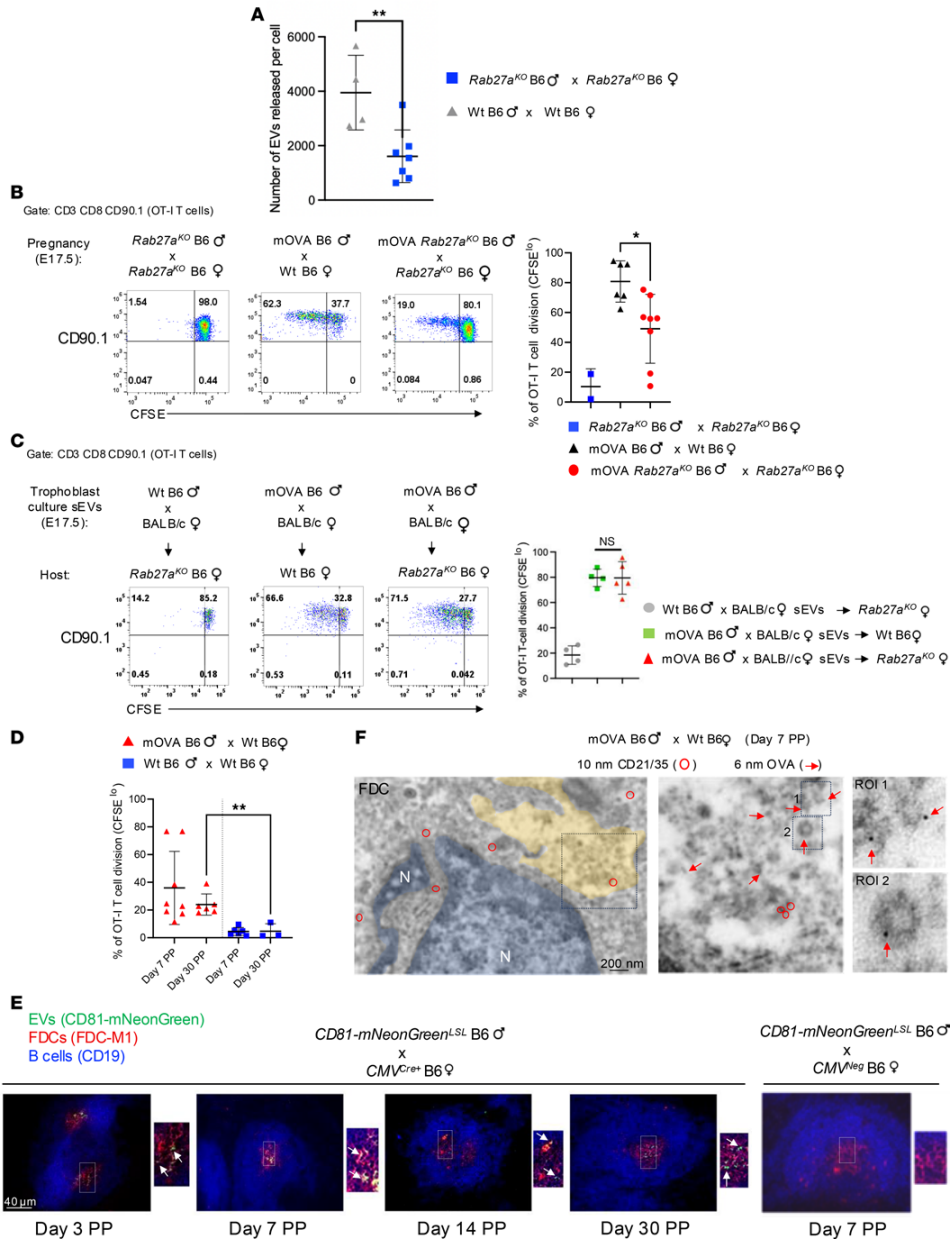
Reduction of release of fetoplacental sEVs decreases maternal T cell recognition of paternal Ag. (**A**) Assessment by NTA of sEVs per trophoblast cell in supernatants of cultures of B6 mouse trophoblasts, WT or *Rab27a^KO^*. Each dot represents sEVs from a different culture. (**B**) Proliferation of CFSE-labeled OT-I CD8^+^ T cells (CD90.1) in spleens of WT or *Rab27a^KO^* B6 females (CD90.2) mated with mOVA B6 or mOVA *Rab27a^KO^* B6 males. OT-I cells were i.v. injected on E15.5 and splenocytes analyzed by FACS on E17.5. Dot plot (right): percentages of dividing OT-I cells, where each dot represents 1 mouse. (**C**) Division of CFSE-labeled OT-I cells (CD90.1) in spleens of WT or *Rab27a^KO^* B6 virgin females injected i.v. with sEVs from trophoblast cultures of E17.5 placentas from BALB/c females impregnated with mOVA B6 males. Dot plot (right): percentages of proliferating OT-I cells, where each dot represents 1 mouse. Splenocytes were analyzed by FACS 2 days after transfer of OT-I cells and injection of trophoblast sEVs. (**D**) Percentages of proliferation of CFSE-labeled OT-I cells (CD90.1) in spleens of B6 females impregnated with WT or mOVA B6 males, assessed by FACS on successive days PP. Each dot represents 1 mouse. (**E**) Microscopy of CD81-mNeonGreen in FDCs in B cell follicles on cryosections of spleens of *CMV^Cre/+^* or *CMV^–^* B6 females, impregnated with Exomap1 B6 males and analyzed PP. Original magnification, ×200, ×400. (**F**) IEM showing OVA-bearing sEVs in maternal CD21/35^+^ FDCs on an ultrathin cryosection of a spleen of a WT B6 female impregnated with a mOVA B6 male and analyzed on day 7 PP. Representative of 2 spleens analyzed. Original magnification, ×20,000, ×80,000. In **A**, comparisons by 2-tailed Student’s test. In **B**–**D**, comparisons by 1-way ANOVA with multiple comparisons. Error bars: means ± SD. *P < 0.05, **P < 0.01.

To validate the link between systemic release of sEVs by the conceptus and detection of fetoplacental Ags by maternal T cells in the maternal spleen in vivo, we used a pregnancy model deficient in Rab27a, a protein previously reported to be critical for exosome release in several cell types (49). Indeed, we verified that primary cultures of *Rab27a^–/–^* B6 mouse trophoblast released approximately 70% fewer sEVs than WT B6 trophoblast cultures (Figure 9A). *Rab27a^–/–^* B6 females were impregnated by Act-mOVA *Rab27a^–/–^* B6 males, and on E15.5 they were i.v. injected with CFSE-labeled CD90.1 OT-I T cells. Analysis of the maternal spleens by FACS on E17.5 revealed that OT-I cells divided significantly less compared with those transferred to control WT B6 females impregnated by Act-mOVA males (Figure 9B).

Lack of Rab27a in DCs has been shown to limit delivery of the NADPH oxidase NOX2 in phagosomes, increasing phagosome acidification and Ag degradation, which could affect OVA crosspresentation by CD8^+^ T cells (50). However, there was no significant difference in OT-I proliferation between WT and *Rab27a^–/–^* B6 virgin female mice i.v. injected with CFSE-labeled OT-I cells and then exposed to trophoblast sEVs carrying mOVA and injected i.v. (Figure 9C). This indicates that Rab27a deficiency in the host’s APCs, or maternal APCs in our pregnancy model, did not decrease presentation of mOVA-derived peptides to the OT-I cells. In summary, reduction of sEVs released by the fetoplacental unit directly correlates with decreased proliferation of CD8^+^ T cells specific for conceptus-derived Ag.

### Retention of fetoplacental sEVs in the maternal spleen.

Next, we assessed how long conceptus-derived sEVs and the paternal Ags they carry are retained postpartum (PP) by the maternal immune cells in the maternal spleen. PP retention of paternal mOVA Ag by the mother’s spleen was assessed by transferring CFSE-labeled CD90.1 OT-I CD8^+^ T cells in WT CD90.2 B6 females previously impregnated by Act-mOVA B6 or by control WT B6 males. OT-I cell division assessed by CFSE dilution by FACS 2 days after OT-I cell administration revealed that OT-I cells proliferated up to a follow-up of 30 days PP, with decreasing percentages of dividing cells over time (Figure 9D). Microscopy analysis of spleens of *CMV^Cre+^* B6 females impregnated by Exomap1 B6 males revealed CD81-mNeonGreen in maternal FDC networks up to 30 days PP (Figure 9E), and IEM of spleens B6 females impregnated with mOVA B6 males verified the presence of sEVs carrying OVA in maternal FDCs 7 days PP (Figure 9F). Thus, fetoplacental sEVs are retained PP by maternal immune cells in SLTs.

### Human trophoblast sEVs target human APCs in the spleen.

**Figure 10:**
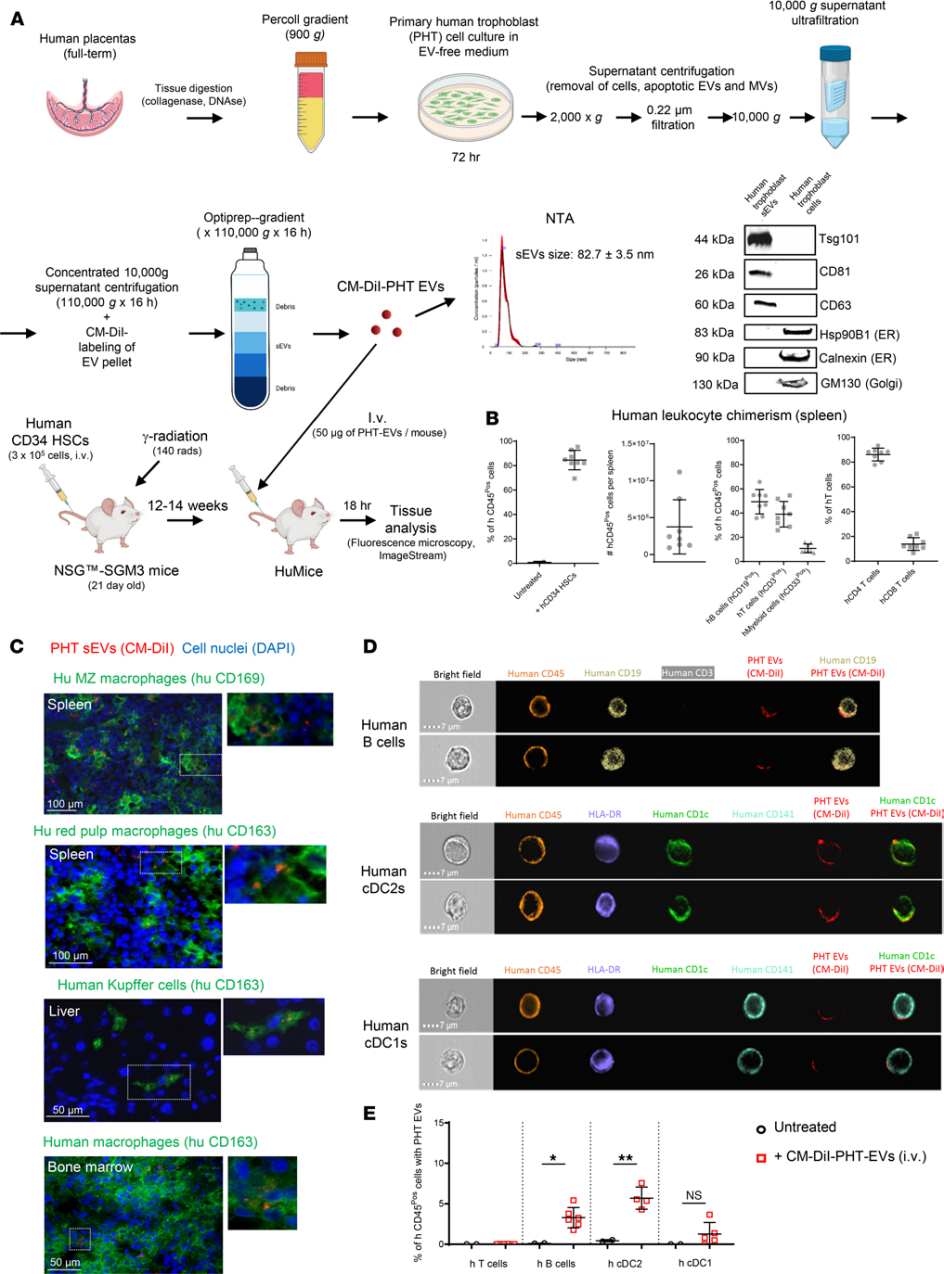
Human trophoblast sEVs are captured by human immune cells in vivo. (**A**) Diagram depicting the generation of PHT cultures and purification of sEVs from PHT culture supernatants. The CM-DiI–labeled PHT-derived sEVs were i.v. injected in huMice, and the traffic of the injected EVs to spleen, bone marrow, liver, lung, and thymus was analyzed 18 hours later. (**B**) Percentages of human leukocyte chimerism in huMice analyzed in the spleen by flow cytometry at the endpoint of the experiments (14–16 weeks after injection of human CD34 hematopoietic stem cells). Each dot represents 1 huMouse. (**C**) Detection by fluorescence microscopy in human macrophages of spleen, liver, and bone marrow of CM-DiI (red) PHT sEVs injected i.v. in huMice. Representative of 6 huMice. Original magnification, ×200. (**D**) Representative ImageStream images of human B cells, cDC2s, and cDC1s carrying CM-DiI PHT sEVs, likely as sEV clusters detectable by the ImageStream analyzer. Original magnification, ×60, out of 20,000 cells analyzed per huMouse spleen. ImageStream analysis was done 18 hours after i.v. injection of the sEVs. (**E**) Pooled data from the ImageStream analysis shown in **D** with percentages of human T cells, B cells, and cDCs with CM-DiI content in splenocytes of huMice untreated or i.v. injected with CM-DiI PHT sEVs. Each dot represents 1 huMouse. In **E**, comparisons by 2-tailed Student’s test. Error bars: means ± SD. *P < 0.05, **P < 0.01.

To evaluate if our findings have a correlation in human pregnancy, we investigated whether sEVs released by the human placenta in the maternal peripheral blood are captured in vivo by human leukocytes in the host’s lymphoid tissues. We conducted this in vivo with a human surrogate model in which sEVs purified from culture supernatants of primary human trophoblast (PHT) cells were labeled red with CM-DiI dye and injected i.v. in humanized mice (huMice) (Figure 10A). The size of the PHT EVs was measured by nanoparticle tracking analysis (Figure 10A). The presence of the EV-associated markers Tsg101, CD63, and CD81 and absence of the EV exclusion markers Hsp90B1 (ER), calnexin (ER), and GM130 (Golgi apparatus) were assessed by Western blot (Figure 10A). The huMice had an average of 80% ± 7.9% of human chimeric leukocytes at the time of the experiments (Figure 10B). Eighteen hours after sEV injection, analysis by microscopy of huMice tissues revealed CM-DiI content in human CD169^+^ MZ macrophages and human CD163^+^ red pulp macrophages in the spleens and in human CD163^+^ macrophages of liver and bone marrow (Figure 10C). Importantly, the Abs against human CD169 and CD163 used did not cross-react with their mouse analogs, which validates that the human PHT-derived sEVs were indeed captured by human leukocytes and not by residual macrophages of mouse origin (Supplemental Figure 7A). ImageStream analysis of single-cell suspensions of splenocytes of huMice injected systemically 18 hours prior with PHT-derived sEVs labeled with CM-DiI showed the presence of CM-DiI in dotted areas on human B cells, human cDC1s (HLA-DR^+^CD141^+^CD1c^–^), and human cDC2s (HLA-DR^+^CD141^–^CD1c^+^) and its absence on human T cells (Figure 10, D and E, and Supplemental Figure 7, B and C). We were unable to analyze traffic of the PHT sEVs to human FDCs in SLTs because huMice do not generate human FDCs. Thus, the human trophoblasts release sEVs that, through peripheral blood, reach human APCs and macrophage subsets in the spleen and human macrophages in liver and bone marrow.

## Discussion

Multiple immunoregulatory mechanisms operate at the fetomaternal interface and in maternal lymphoid tissues to silence the innate and adaptive immunological attacks on the fetus while preserving the capacity of the mother to mount effector responses against pathogens (51–57).

However, an unanswered question in the immunology of pregnancy is how fetoplacental Ags are relayed to maternal APCs in the mother’s SLTs, in a manner that the conceptus-derived native Ags can be internalized, processed, and presented as Ag peptides in maternal MHC molecules to maternal T cells. This mechanism is of clinical relevance, since its dysregulation may lead to immune-mediated pregnancy disorders. The physical properties in which fetoplacental Ags are shed systemically could be key to rendering fetoplacental Ags nonimmunogenic against the conceptus or, alternatively, to channel the Ags to a maternal APC subset(s) that controls the maternal T cell response against the fetoplacental unit (7). Ag transfer could occur through fetoplacental microchimeric cells that migrate to maternal SLTs. However, during gestation, fetoplacental cells have been detected at extremely low numbers in maternal blood and peripheral organs (15, 16, 58). Alternatively, embryonic and paternal Ags could be delivered systemically to the maternal SLTs via a cell-free platform by blood circulating free Ag proteins or transported in EVs.

Previous work by others and us has shown that human trophoblast sEVs transport placenta-specific microRNAs that confer viral resistance to the EV-acceptor cells and that fetoplacental EVs play multiple roles in the pathogenesis of pregnancy disorders (34, 36, 59). Although the surrogate paternal Ag mOVA has been reported on sEVs from plasma of pregnant mice, and release of fetoplacental sEVs in maternal blood has been suggested as a mechanism of transfer of paternal and placental Ags to the mother’s SLTs, to our knowledge this idea has not been formally investigated (7, 10).

To determine the anatomical location of the trophoblast cells that release sEVs in relation to the maternal blood spaces, we developed mouse models in which sEVs generated by the fetoplacental unit are identified by their expression of fluoroprobes linked to tetraspanins enriched in sEVs shed from the cell surface (i.e., CD81) or generated in endosomal compartments (i.e., CD63). In our system, mOVA was targeted to the plasma and sEV membranes via the transmembrane sequence of the mouse H-2D^b^ molecule (10, 60). It is unknown to which extent free mOVA is released from trophoblast cells or their sEVs by proteolytic cleavage. This effect or the presence of nonpelleted sEVs could explain the presence of OVA in the 10,000*g* and 100,000*g* centrifugation supernatants of plasma of pregnant mice and trophoblast culture supernatants. To our knowledge, it is unknown whether OVA or the H-2D^b^ transmembrane domain in mOVA are targets of ADAM10 and ADAM17, 2 metalloproteinases described in sEVs (61, 62).

Previous studies in pregnant mice demonstrated that the surrogate paternal Ag mOVA, released systemically, is detectable by microscopy on tissue sections in FDCs of the maternal spleen and lymph nodes (LNs) (1, 47). The format in which mOVA is retained by FDCs and if other maternal immune cells in SLTs capture fetoplacental Ags in amounts previously undetected by fluorescence microscopy have not been explored. Our analysis by IEM indicates that maternal FDCs concentrate mOVA through internalization of clusters of sEVs bearing mOVA, though other alternative mechanisms of retention of conceptus-derived mOVA by FDCs cannot be ruled out. In this regard, human tonsil FDCs have been shown to bind endogenous exosomes likely secreted by neighboring B cells (63). FDCs internalize HIV in cycling endosomes (64), an interesting fact considering enveloped viruses share biogenesis, size, and mechanisms of adsorption and cell entry with sEVs (65). In addition, our ultrastructural analysis of the maternal spleen during gestation also detected conceptus-derived sEVs carrying mOVA crossing MZ CD169^+^ macrophages by transcytosis and in proximity to cDCs. Interestingly, by a similar mechanism, graft-derived sEVs carrying donor allogeneic Ags cross recipients’ MZ macrophages in the spleen and subcapsular sinus CD169^+^ macrophages in graft-draining LNs (22).

The finding that sEVs bearing mOVA reach maternal immune cells in the spleen suggested that the vesicles are transferred locally from fetoplacental cells that traffic to the mother’s spleen or systemically via maternal blood, since the spleen lacks afferent lymphatics. However, migratory fetoplacental cells secreting mScarlet-CD63 sEVs and identifiable by their BFP-tagged nuclei were not detected in the mother’s spleen. By contrast, conceptus-derived sEVs bearing mOVA were found in plasma taken between E12.5 and E17.5 from WT BALB/c female mice impregnated with Act-mOVA B6 males, indicating that fetoplacental sEVs are disseminated mainly via maternal blood. Accordingly, i.v. administration of sEVs purified from cultures of CD81-mNeonGreen mouse trophoblasts recapitulate the relay of endogenous CD81-mNeonGreen sEVs to the maternal immune cells in spleens of *CMV^Cre/+^* B6 females impregnated with *tdTomato^LSL^ mNeonGreen* B6 males.

We used primary mouse trophoblast cultures to generate sufficient trophoblast sEVs to test, in vivo, their capacity to systemically deliver the paternal Ag mOVA to APCs in the spleen of virgin mice and analyze the subsequent CD8^+^ T cell response ex vivo. Although a single bolus i.v. injection of trophoblast sEVs bearing mOVA in virgin female mice led to OT-I cell proliferation, this was associated with lesser activation, increased percentage of apoptotic cells, and decreased expression of the effector markers granzyme B and IFN-γ, as compared with controls in which optimal T cell stimulation was induced. These findings mimic, to some extent, the response that takes place on i.v. transferred OT-I cells or OVA-specific endogenous CD8^+^ T cells during gestation in WT B6 females impregnated by Act-mOVA B6 males (1, 7, 9).

Previous studies in mice revealed that primary pregnancies elicit expansion of fetal OVA-specific endogenous polyclonal T cells that are hypofunctional and with increased expression of T cell exhaustion markers (11–14). These exhausted T cells remained elevated after parturition and increased in percentage in secondary pregnancies sired by OVA-expressing males (11). It is likely that in our model, the transient stimulation of the host’s immune system by a single i.v. injection of trophoblast sEVs carrying mOVA was insufficient to recapitulate the prolonged exposure to fetoplacental Ags of the mother’s T cells during gestation. This could also explain why, in our hands, we could not detect an increase of expression of Lag3, TIM-1, and PD-1 on the adoptively transferred OT-I cells that proliferated in response to systemically infused trophoblast sEVs bearing mOVA.

In the mOVA pregnancy model used in the present study, others have shown that the maternal cDCs are the main APCs that present the OVA-peptides to OT-I cells in the mother’s spleen (66). Accordingly, using 2 mouse models that enable us to track the fate of fetoplacental sEVs in the mother’s SLTs, we found that the mother’s cDCs in the spleen capture CD81-mNeonGreen sEVs or mScarlet-CD63 sEVs released by the fetoplacental unit. It is known that DCs efficiently process Ags carried by internalized sEVs into peptides that are presented to T cells in the context of the MHC molecules expressed by the acceptor cells (67, 68).

A caveat of our study is that because of technical reasons, we compared the effect of a single injection of trophoblast sEVs in virgin females with the continuous release of sEVs by the fetoplacental unit during gestation. Thus, our mouse model did not allow us to test for OT-I cell exhaustion PP because the relay of mOVA to the maternal spleen was transient, unlike the continuous delivery of conceptus-derived mOVA to the mother’s spleen during middle and late gestation. Another limitation is that the OT-I cell response to the infused trophoblast sEVs was analyzed in virgin females, in which only the intrinsic effect(s) of the trophoblast sEVs on the OT-I cell response can be tested. Moreover, the high fixed affinity of the TCR-transgenic OT-I cells may not exactly reproduce the response of endogenous polyclonal CD8^+^ T cells throughout gestation.

A relevant question is whether the trophoblast sEVs carry immunoregulatory mediators that affect the APC function of the maternal immune cells targeted by the vesicles in SLTs. Comparative analysis by high-resolution LC-MS of trophoblast sEVs and their parent trophoblast cells revealed that several proteins with antiinflammatory or immunosuppressive function are highly enriched in trophoblast sEVs. Importantly, some of these proteins, such as pigment epithelium-derived factor and neuropilin-1, suppress DC maturation and promote Treg function, while others, such as apolipoproteins M and E, thrombospondins 1 and 2, antithrombin III, and prolactins, prevent activation of NF-κB and secretion of IL-1β, IL-6, and TNF-α via different pathways (Supplemental Table 2). Other proteins significantly enriched in trophoblast sEVs included mannose receptor 2, carcinoembryonic Ag, insulin-like growth factor receptor 2, and galectin-3–binding protein, which via different mechanisms induce DCs to secrete IL-10, suppressing the release IL-12 and therefore preventing type 1–biased immunity. Some of these proteins have been shown to inhibit CD8^+^ T cells and suppress the immunostimulatory function of NK cells (Supplemental Table 2). Interestingly, neuropilin-1 and apolipoprotein E have recently been described in EVs released by the human syncytiotrophoblast (69, 70). Thus, trophoblast sEVs constitute an efficient vehicle to deliver fetoplacental Ags together with antiinflammatory and immunosuppressive mediators to target maternal immune cells in SLTs. This phenomenon mimics, to some extent, the constant relay of self-Ags from peripheral tissues to immature/semimature DCs in SLTs, which participates in maintenance of T cell peripheral tolerance in steady-state conditions (71).

The role of sEVs as a platform to deliver fetoplacental Ags to the maternal APCs in SLTs was validated in pregnancies carrying concepti deficient in Rab27a, from which primary cultures of trophoblasts secreted significantly lower numbers of sEVs compared with WT controls (Figure 9A). The role of Rab27a in release of sEVs remains controversial. *Rab27a* deletion, *Rab27a* mRNA knockdown, or elevated degradation of Rab27a protein reduces sEV release in vitro and in vivo (49, 72, 73); nevertheless, in certain cell lines, lack of Rab27a does not decrease sEV secretion (43). Cell type–specific differences or compensatory mechanisms may explain these differences. Rab27a may also have functions other than sEV release, including control of intracellular membrane trafficking (74–76).

It is well established in vaccine design that nanoparticles carrying multiple copies of the immunogen deliver Ags to SLTs more efficiently than monomeric Ag and that particles in the range of 50–100 nm in diameter are the most effective (77, 78). Thus, fetoplacental sEVs (~100 nm) are within the optimal size for delivery of conceptus Ags to maternal SLTs and can carry multiple copies of the same Ag on their surface or in the vesicle lumen. Indeed, trophoblast sEVs administered i.v. induced more OT-I cell proliferation in the spleen than that elicited by lysed sEVs. Also, fetoplacental Ags on the surface of sEVs can be recognized in their native form by maternal B cells. Fetoplacental sEVs were detected in the maternal spleen up to 30 days PP, which agrees with previous data on retention of conceptus Ags by maternal SLTs (9). PP presentation of fetoplacental sEVs held in the mother’s SLTs may contribute to maternal sensitization against conceptus Ags after delivery.

Further studies will be necessary to determine if sEVs shed by the conceptus function simply as vehicles to deliver fetoplacental Ags to the mother’s SLTs in the pro-tolerogenic environment of gestation. Alternatively, conceptus-derived EVs could be intrinsically immunosuppressive and deliver fetoplacental Ag in combination with mediators that downregulate or bias the Ag-presenting ability of maternal APCs. In this regard, we found that mOVA on sEVs from trophoblast cultures and plasma of pregnant mice were glycosylated with N-linked glycans. Trophoblast-derived mOVA bearing N-glycosylated α2,6 and α2,3 sialic acids have been recently shown to suppress maternal B cells by binding the inhibitory receptor CD22 and Siglec-G (66), though the relevance of this phenomenon is not well understood, since pregnancy also induces humoral sensitization in humans and mice (14).

Importantly, the ability of sEVs secreted by the human placenta to reach human immune cells in SLTs was validated in huMice, a model in which PHT sEVs injected i.v. targeted human cDC1s, cDC2s, and B cells in the spleen. This agrees with our findings in pregnant mice and with the previous observation in mice in which cDCs and B cells are the maternal APCs that present fetoplacental mOVA Ag to CD8^+^ and CD4^+^ T cells, respectively (66). We were unable to analyze the relay of human placenta sEVs to human FDCs, since FDCs are of stromal origin and therefore do not develop in huMice generated with human CD34^+^ hematopoietic stem cells.

In conclusion, our findings unveil an sEV-based mechanism for the fetoplacental unit to communicate with maternal immune cells in the mother’s SLTs. Importantly, these sEVs carry fetoplacental Ags that are processed and presented by the mother’s APCs to T cells. Delivery of multiple copies of Ags on nanoparticles with the size of naturally occurring sEVs is an efficient system to deliver Ag to SLTs (77, 78). Here, we showed that the fetoplacental unit does precisely that by releasing sEVs systemically. Our study does not preclude the relay of fetoplacental Ags via other cell-free pathways, since it is likely that not all embryonic or parental Ags are sorted into EVs. Changes in the Ag repertoire or content of immunoregulatory mediators in fetoplacental EVs could participate in the pathogenesis of immune-mediated pregnancy disorders.

## Methods

Further information can be found in Supplemental Methods. Resources can be found in Supplemental Table 3.

### Sex as a biological variable.

In vivo pregnancy experiments were carried out on female mice impregnated by male mice. Primary cultures of mouse trophoblasts were generated from placentas of male and female concepti. All human donors of cord blood were anonymous (i.e., ethnicity and age are unknown).

### Mice and reagents.

NSG-SGM3, Act-mOVA C57BL/6 (mOVA B6), B6.CTg (CMV-Cre)1Cgn/J (CMV-Cre), and BALB/c mice were purchased from Charles River Laboratories. CD90.1 Rag1^–/–^ OT-I B6 (OT-I) were provided by F. Lakkis (University of Pittsburgh). Exomap1 B6 mice were generated by The Johns Hopkins University. *Rab27a^KO^* B6 mice were generated by The Scripps Research Institute. Mice were bred and maintained in the pathogen-free animal facility of the University of Pittsburgh School of Medicine.

### Generation of primary cultures of mouse trophoblast cells.

Placentas were removed with fine forceps from euthanized pregnant mice (E12.5–E17.5) and immediately placed in ice-cold PBS. Ten to 25 placentas were minced and digested with Medium-199 supplemented with sodium bicarbonate (0.01 M), HEPES, Liberase (0.25 Wunsch units/mL), and DNase I (0.2 mg/mL), for 1 hour at 37°C. After digestion, cell suspensions were passed through a 100 μm strainer and centrifuged at 500*g*, at 4°C, for 5 minutes. Supernatant was removed and the cell pellet was resuspended in 5 mL RBC lysis buffer (MilliporeSigma) for 10 minutes at room temperature. RBC lysis buffer was removed by adding an excess of PBS followed by centrifugation at 500*g*, at 4°C, for 5 minutes. Next, the cell pellet was resuspended in 10 mL of ice-cold 0.01 M EDTA/PBS and centrifuged (500*g*, at 4°C, 5 minutes). For isolation of mouse trophoblast cells with a Percoll gradient, the placenta cell suspensions were resuspended in 3 mL of PBS and mixed with 14.4 mL of Percoll, 20 mL of 1× Medium-199, and 1.6 mL of 10× Medium-199, followed by centrifugation at 30,000*g*, at 4°C, for 30 minutes. Trophoblast cells were collected from the gradient and washed with 30 mL of ice-cold PBS and centrifuged at 500*g*, at 4°C, for 5 minutes. Primary mouse trophoblast cells were cultured on Matrigel-coated, 6-well plates (10,000,000 cells/well) for 2 days in NCTC-109 medium supplemented with 5% EV-free FBS.

### Statistics.

GraphPad Prism was used for statistical analyses. Results are expressed as means ± SD. Comparisons between 2 groups were performed with 2-tailed Student’s *t* test. Comparisons of more than 2 groups were performed with 1-way ANOVA, followed by Tukey-Kramer multiple comparisons test. In all experiments, *P* ≤ 0.05 was considered significant.

### Study approval.

Animal care and handling were performed in accordance with institutional guidelines and procedures approved by the IACUC, University of Pittsburgh, Pittsburgh, Pennsylvania, USA, protocol numbers 22091764 and 23043086. Whole placentas and cord blood from the umbilical cord and placenta of anonymous, healthy pregnant women were used according to the guidelines of the IRB of the University of Pittsburgh, which waived the need for informed consent. Studies on huMice were approved by the IACUC (protocol number 20067566) and the IRB (number PRO13120232) of the University of Pittsburgh.

### Data availability.

Data are available in the Supporting Data Values XLS file or from the corresponding author upon request.

## Author contributions

JSP, YS, and AEM conceived the study. AEM and YS supervised the study. JSP, ATL, WJS, and AEM performed in vitro and in vivo experiments. JSP, WJS, MLGS, DBS, SCW, and AEM performed imaging. JSP and AEM analyzed data. SJG, GC, and SDC provided resources. JSP, YS, and AEM developed methodology. AEM and YS acquired funding. JSP, YS, and AEM wrote the manuscript.

## Supplementary Material

Supplemental data

Unedited blot and gel images

Supporting data values

## Version 1 05/22/2025

Electronic publication

## References

[1] Erlebacher A (2007). Constraints in antigen presentation severely restrict T cell recognition of the allogeneic fetus. J Clin Invest.

[2] Moldenhauer LM (2009). Cross-presentation of male seminal fluid antigens elicits T cell activation to initiate the female immune response to pregnancy. J Immunol.

[3] Aluvihare VR (2004). Regulatory T cells mediate maternal tolerance to the fetus. Nat Immunol.

[4] Rowe JH (2012). Pregnancy imprints regulatory memory that sustains anergy to fetal antigen. Nature.

[5] Rowe JH (2011). Foxp3(+) regulatory T cell expansion required for sustaining pregnancy compromises host defense against prenatal bacterial pathogens. Cell Host Microbe.

[6] Samstein RM (2012). Extrathymic generation of regulatory T cells in placental mammals mitigates maternal-fetal conflict. Cell.

[7] Tay CS (2013). Cis-acting pathways selectively enforce the non-immunogenicity of shed placental antigen for maternal CD8 T cells. PLoS One.

[8] Taglauer ES (2009). Maternal PD-1 regulates accumulation of fetal antigen-specific CD8^+^ T cells in pregnancy. J Reprod Immunol.

[9] Barton BM (2017). Pregnancy promotes tolerance to future offspring by programming selective dysfunction in long-lived maternal T cells. J Leukoc Biol.

[10] Jasti S (2017). Immune response to a model shared placenta/tumor-associated antigen reduces cancer risk in parous mice. Biol Reprod.

[11] Kinder JM (2020). CD8^+^ T cell functional exhaustion overrides pregnancy-induced fetal antigen alloimmunization. Cell Rep.

[12] Lewis EL (2022). NFAT-dependent and -independent exhaustion circuits program maternal CD8 T cell hypofunction in pregnancy. J Exp Med.

[13] Pollard JM (2024). Pregnancy dedifferentiates memory CD8^+^ T cells into hypofunctional cells with exhaustion-enriched programs. JCI Insight.

[14] Suah AN (2021). Pregnancy-induced humoral sensitization overrides T cell tolerance to fetus-matched allografts in mice. J Clin Invest.

[15] Herzenberg LA (1979). Fetal cells in the blood of pregnant women: detection and enrichment by fluorescence-activated cell sorting. Proc Natl Acad Sci U S A.

[16] Bonney EA, Matzinger P (1997). The maternal immune system’s interaction with circulating fetal cells. J Immunol.

[17] Dakic A (2004). Development of the dendritic cell system during mouse ontogeny. J Immunol.

[18] Collins MK (2009). Dendritic cell entrapment within the pregnant uterus inhibits immune surveillance of the maternal/fetal interface in mice. J Clin Invest.

[19] Morelli AE, Sadovsky Y (2022). Extracellular vesicles and immune response during pregnancy: a balancing act. Immunol Rev.

[20] Robbins PD, Morelli AE (2014). Regulation of immune responses by extracellular vesicles. Nat Rev Immunol.

[21] Buzas EI (2023). The roles of extracellular vesicles in the immune system. Nat Rev Immunol.

[22] Zeng F (2021). Graft-derived extracellular vesicles transported across subcapsular sinus macrophages elicit B cell alloimmunity after transplantation. Sci Transl Med.

[23] Salomon C (2017). Placental exosomes as early biomarker of preeclampsia: potential role of exosomal microRNAs across gestation. J Clin Endocrinol Metab.

[24] Sabapatha A (2006). Specific isolation of placenta-derived exosomes from the circulation of pregnant women and their immunoregulatory consequences. Am J Reprod Immunol.

[25] Germain SJ (2007). Systemic inflammatory priming in normal pregnancy and preeclampsia: the role of circulating syncytiotrophoblast microparticles. J Immunol.

[26] Holder BS (2012). Immune cell activation by trophoblast-derived microvesicles is mediated by syncytin 1. Immunology.

[27] Dragovic RA (2013). Multicolor flow cytometry and nanoparticle tracking analysis of extracellular vesicles in the plasma of normal pregnant and pre-eclamptic women. Biol Reprod.

[28] Tannetta DS (2013). Characterisation of syncytiotrophoblast vesicles in normal pregnancy and pre-eclampsia: expression of Flt-1 and endoglin. PLoS One.

[29] Nguyen SL (2019). Quantifying murine placental extracellular vesicles across gestation and in preterm birth data with tidyNano: a computational framework for analyzing and visualizing nanoparticle data in R. PLoS One.

[30] Sheller-Miller S (2019). Exosomes cause preterm birth in mice: evidence for paracrine signaling in pregnancy. Sci Rep.

[31] Ouyang Y (2016). Isolation of human trophoblastic extracellular vesicles and characterization of their cargo and antiviral activity. Placenta.

[32] Chamley LW (2011). Trophoblast deportation: just a waste disposal system or antigen sharing?. J Reprod Immunol.

[33] Sarker S (2014). Placenta-derived exosomes continuously increase in maternal circulation over the first trimester of pregnancy. J Transl Med.

[34] Mitchell MD (2015). Placental exosomes in normal and complicated pregnancy. Am J Obstet Gynecol.

[35] Holland OJ (2012). Minor histocompatibility antigens are expressed in syncytiotrophoblast and trophoblast debris: implications for maternal alloreactivity to the fetus. Am J Pathol.

[36] Nair S, Salomon C (2018). Extracellular vesicles and their immunomodulatory functions in pregnancy. Semin Immunopathol.

[37] Mincheva-Nilsson L (2021). Immunosuppressive protein signatures carried by syncytiotrophoblast-derived exosomes and their role in human pregnancy. Front Immunol.

[38] Tong M (2018). Immunological effects of placental extracellular vesicles. Immunol Cell Biol.

[39] Göhner C (2017). Immune-modulatory effects of syncytiotrophoblast extracellular vesicles in pregnancy and preeclampsia. Placenta.

[40] Favaro RR (2021). Immunomodulatory properties of extracellular vesicles in the dialogue between placental and immune cells. Am J Reprod Immunol.

[41] Powell JS (2022). Small extracellular vesicles from plasma of women with preeclampsia increase myogenic tone and decrease endothelium-dependent relaxation of mouse mesenteric arteries. Pregnancy Hypertens.

[42] Fordjour FK (2023). Exomap1 mouse: a transgenic model for *in vivo* studies of exosome biology. Extracell Vesicle.

[43] Fordjour FK (2022). A shared, stochastic pathway mediates exosome protein budding along plasma and endosome membranes. J Biol Chem.

[44] Enders AC (1965). A comparative study of the fine structure of the trophoblast in several hemochorial placentas. Am J Anat.

[45] Mathieu M (2021). Specificities of exosome versus small ectosome secretion revealed by live intracellular tracking of CD63 and CD9. Nat Commun.

[46] Ai Y (2024). Endocytosis blocks the vesicular secretion of exosome marker proteins. Sci Adv.

[47] McCloskey ML (2011). Acquisition and presentation of follicular dendritic cell-bound antigen by lymph node-resident dendritic cells. J Exp Med.

[48] Kugeratski FG (2021). Quantitative proteomics identifies the core proteome of exosomes with syntenin-1 as the highest abundant protein and a putative universal biomarker. Nat Cell Biol.

[49] Ostrowski M (2010). Rab27a and Rab27b control different steps of the exosome secretion pathway. Nat Cell Biol.

[50] Jancic C (2007). Rab27a regulates phagosomal pH and NADPH oxidase recruitment to dendritic cell phagosomes. Nat Cell Biol.

[51] Mor G (2017). The unique immunological and microbial aspects of pregnancy. Nat Rev Immunol.

[52] Park JE (2020). Prenatal development of human immunity. Science.

[53] Erlebacher A (2013). Mechanisms of T cell tolerance towards the allogeneic fetus. Nat Rev Immunol.

[54] Erlebacher A (2013). Immunology of the maternal-fetal interface. Annu Rev Immunol.

[55] PrabhuDas M (2015). Immune mechanisms at the maternal-fetal interface: perspectives and challenges. Nat Immunol.

[56] Ander SE (2019). Immune responses at the maternal-fetal interface. Sci Immunol.

[57] Megli CJ, Coyne CB (2022). Infections at the maternal-fetal interface: an overview of pathogenesis and defence. Nat Rev Microbiol.

[58] Shao TY (2023). Reproductive outcomes after pregnancy-induced displacement of preexisting microchimeric cells. Science.

[59] Delorme-Axford E (2013). Human placental trophoblasts confer viral resistance to recipient cells. Proc Natl Acad Sci U S A.

[60] Ehst BD (2003). Development of a novel transgenic mouse for the study of interactions between CD4 and CD8 T cells during graft rejection. Am J Transplant.

[61] Groth E (2016). Stimulated release and functional activity of surface expressed metalloproteinase ADAM17 in exosomes. Biochim Biophys Acta.

[62] Stoeck A (2006). A role for exosomes in the constitutive and stimulus-induced ectodomain cleavage of L1 and CD44. Biochem J.

[63] Denzer K (2000). Follicular dendritic cells carry MHC class II-expressing microvesicles at their surface. J Immunol.

[64] Heesters BA (2015). Follicular dendritic cells retain infectious HIV in cycling endosomes. PLoS Pathog.

[65] (2011). Microvesicles and viral infection. J Virol.

[66] Rizzuto G (2022). Establishment of fetomaternal tolerance through glycan-mediated B cell suppression. Nature.

[67] Thery C (2002). Indirect activation of naïve CD4^+^ T cells by dendritic cell-derived exosomes. Nat Immunol.

[68] Morelli AE (2004). Endocytosis, intracellular sorting, and processing of exosomes by dendritic cells. Blood.

[69] Awoyemi T (2022). Neuropilin-1 is uniquely expressed on small syncytiotrophoblast extracellular vesicles but not on medium/large vesicles from preeclampsia and normal placentae. Biochem Biophys Res Commun.

[70] Tersigni C (2022). Syncytiotrophoblast-derived extracellular vesicles carry apolipoprotein-E and affect lipid synthesis of liver cells in vitro. J Cell Mol Med.

[71] Steinman RM, Nussenzweig MC (2002). Avoiding horror autotoxicus: the importance of dendritic cells in peripheral T cell tolerance. Proc Natl Acad Sci U S A.

[72] Song L (2019). KIBRA controls exosome secretion via inhibiting the proteasomal degradation of Rab27a. Nat Commun.

[73] Bauer KM (2022). CD11c^+^ myeloid cell exosomes reduce intestinal inflammation during colitis. JCI Insight.

[74] Izumi T (2021). In vivo roles of Rab27 and its effectors in exocytosis. Cell Struct Funct.

[75] Fukuda M (2013). Rab27 effectors, pleiotropic regulators in secretory pathways. Traffic.

[76] Gerber PP (2015). Rab27a controls HIV-1 assembly by regulating plasma membrane levels of phosphatidylinositol 4,5-bisphosphate. J Cell Biol.

[77] Tokatlian T (2019). Innate immune recognition of glycans targets HIV nanoparticle immunogens to germinal centers. Science.

[78] Zhang YN (2019). Nanoparticle size influences antigen retention and presentation in lymph node follicles for humoral immunity. Nano Lett.

