# Supplementary material for: Fetoplacental extracellular vesicles deliver conceptus-derived antigens to maternal secondary lymphoid tissues for immune recognition
Source: JCI Insight. 2025 May 22;10(10):e186335. doi: 10.1172/jci.insight.186335 (PMC12128977; doi:10.1172/jci.insight.186335)
Supplement: Supplemental data [file jciinsight-10-186335-s164.pdf]

Supplemental Materials for

Fetoplacental extracellular vesicles deliver conceptus-derived antigens to maternal secondary lymphoid tissues for immune recognition

Juliana S. Powell, Adriana T. Larregina, William J. Shufesky, Mara L.G. Sullivan, Donna Beer Stolz, Stephen J. Gould, Geoffrey Camirand, Sergio D. Catz, Simon C. Watkins, Yoel Sadovsky, and Adrian E. Morelli

The PDF file includes:

Supplemental Methods

Supplemental Figure S1. Expression of tdTomato in control mouse trophoblast.

Supplemental Figure S2. Fetoplacental sEVs at the maternal-fetal interface.

Supplemental Figure S3. Detection of fetoplacental-derived CD81-mNeonGreen in the maternal spleen is not caused by trophoblast-derived tissue fragments.

Supplemental Figure S4. Detection of sEVs bearing fetoplacental Ag in maternal immune cells in the spleen.

Supplemental Figure S5. Ultrastructural analysis of localization of mOVA on mouse trophoblast cells.

Supplemental Figure S6. I.v. injection of trophoblast sEVs and T cell exhaustion in the spleen.

Supplemental Figure S7. Primary human trophoblast (PHT) sEVs traffic to huMice tissues.

Supplemental Table S1. sEV-associated proteins enriched in trophoblast sEVs compared to trophoblast cells.

Supplemental Table S2. Proteins detected by LC-MS with anti-inflammatory or immunosuppressive effects significantly enriched in trophoblast sEVs compared with their trophoblast parent cells.

References for Supplemental Methods and Supplemental Tables S1 and S2.

Supplemental Table S3. Resources.

Supplemental Methods

Generation of conditional transgenic mice

Nuclear-BFP / mScarlet-CD63^{LSL} B6 mice were generated by the Transgenic and Gene Targeting Core of the University of Pittsburgh. A targeting vector was designed as a single open reading frame with a *loxP*-flanked stop sequence (puromycin *N*-acetyltransferase with a SV40 polyA signal) followed by *Homo sapiens H2B* clustered histone 11 (H2BC11) fused to mTagBFP2 cDNA, and mScarlet-I fused *to Mus musculus* CD63 cDNA. The two fusion proteins were linked by a T2A self-cleaving peptide. The cassette was knocked in the *Rosa26* locus and its expression is driven by the strong synthetic CAG promoter. A mixture of the linearized vector, sgRosa26-1 and EnGen Cas9 protein (New England Biolabs) was microinjected in the pronuclei of B6 zygotes, and embryos developed to the 2-cell stage were transferred to the oviducts of pseudo-pregnant B6 females. Potential founders were identified in the pups by PCR-genotyping. Four sets of primers were needed to confirm the lineage. The first set was designed to confirm that the construct was within the *Rosa26* locus (*Rosa26* F06 forward primer = 5'-CTCGGCTAGGTAGGGGATCG-3', *Rosa26* R06 reverse primer = 5'-TGGACTACTGCGCCCTACA-3'). Another 3 pairs of primers were used to check that the LoxP - H2BC11 - mTagBFP2 - mScarlet-I - CD63 cDNA polyA tail fragment of the construct was present in the correct orientation (Set 1: hPACs1 forward primer = 5'-TTGTGGTTTGTCCAAACTCAT-3' and mTagBFP R1 reverse primer = 5'-TTCTTCTGCATAACTGGTCCGT-3', Set 2: mTagBFP F1 forward primer = 5'-GCCTTATGAGGGAACCCAGAC-3' and CD63 R1 reverse primer = 5'-CACCCACTGCAATGATGACCA-3' Set 3: CD63 F1 forward primer = 5'-TTGGTGTAGCGGTTCAGGTTGT-3' and *Rosa26* R05 reverse primer 5'-CAGAAGACTCCCGCCCATCT-3'), Founders were backcrossed with wt B6 mice for 4 generations and then *nuclear-BFP / mScarlet-CD63^{LSL}* B6 mice were crossed between them to generate homozygous animals. A pair of PCR primers was used to confirm homozygosity by testing the absence of remnant wt *Rosa26* genes (*Rosa26* F02 forward primer = 5'CGTTTCCGACTTGAGTTGCC-3', *Rosa26* R02 = 5'-ACTCGGGTGAGCATGTCTTT-3'). PCR reactions were done with the Q5 High-Fidelity 2X Master Mix (New England Biolabs).

Generation of huMice

CD34 hematopoietic stem cells (HSCs) were isolated from cord blood from the umbilical cord and placenta of healthy pregnant women according to the guidelines of the Institutional Review Board (IRB) of the University of Pittsburgh. Heparinized cord blood was diluted (1:1) with phosphate-buffered saline (PBS), and peripheral blood mononuclear cells (PBMCs) were purified with two consecutive discontinuous Ficoll-Paque (1.077 g/ml) density gradients (900*g* for 30 min at 18° to 20°C). Human HSCs were purified from PBMCs by magnetic sorting with CD34 Ab microbeads (Miltenyi Biotec) and cryopreserved in liquid nitrogen until use. CD34 HSC purity by flow cytometry analysis was ≥ 90% with less than 1% of CD3 T cells. To reconstitute NSG-SGM3 mice, CD34 HSCs were thawed, washed in PBS, and cultured in RPMI 1640 supplemented with 10% fetal bovine serum (FBS) for 1 hour at 37°C. A minimum of 3 x 10^5 HSCs in 300 µl of PBS were i.v. injected in 3 wk-old NSG-SGM3 mice, γ-irradiated (140 cGy) 2 hours prior. huMice were treated with Neomycin and Polymycin B in the drinking water during the first week after irradiation. Percentages of human leukocyte chimerism were determined by flow cytometry analysis in peripheral blood 12 weeks after HSC injection, and in the spleen at the end of the experiments (14 to 16 weeks). huMice were left untreated or injected i.v. with CM-DiI-labeled human PHT-derived sEVs (50 µg / huMice). Eighteen hours later, mice were euthanized for analysis of traffic of the injected EVs in spleen, bone marrow, liver, thymus and lung.

Immunofluorescence microscopy

Mouse and huMice tissue fragments were embedded in Optimal Cutting Temperature Compound (OCT), snap-frozen in 2 methyl-butane pre-chilled in liquid nitrogen and stored at -80°C until use. Frozen tissues were sectioned (8 µm) with a cryostat and mounted on slides pre-treated with Vectabond (Vector Laboratories). Tissue sections were fixed in 4% paraformaldehyde (PF) / PBS (15 min, RT), and treated with 5% normal goat or horse serum followed by the avidin/biotin blocking kit (Vector).

Cryosections of placentas of CMV-Cre or wt B6 female mice mated with Exomap1 B6 male mice were incubated with: (i) biotin-pan-keratin Ab followed by AF647-streptavidin (1:400); (ii) desmin goat IgG polyclonal Ab followed by AF647-anti-goat IgG (1:400); (iii) purified CD31 Ab followed by AF647-anti-rat IgG (1:400); or (iv) biotin-CD45 Ab followed by AF647-streptavidin (1:400). Cryosections of spleens of CMV-Cre or wt B6 female mice impregnated by Exomap1 B6 male mice were incubated with the following reagents: (i) purified FDC-M1 rat IgG Ab, followed by AF555-anti-rat IgG (1:400), blocked with rat irrelevant IgG, followed by AF647-CD19 Ab; (ii) purified CD169 or F4/80 Ab followed by AF555-anti-rat IgG; (iii) purified CD11c hamster IgG Ab and biotin-CD3 Ab followed by Cy3-anti-armenian hamster IgG (1:400) and AF647-streptavidin (1:400), or (iv) purified FDC-M1 rat IgG Ab and biotin-pan-keratin Ab followed by AF555-anti-rat IgG (1:400) and AF647-streptavidin (1:400).

Cryosections of placentas of wt BALB/c female mice mated with mOVA or wt B6 male mice were incubated with: (i) purified chicken OVA rabbit IgG polyclonal Ab, biotin-pan-keratin Ab and purified CD31 rat IgG Ab, followed by AF488-anti rabbit IgG (1:400), Cy3-streptavidin (1:1000) and AF647-anti-rat IgG (1:400). Cryosections of spleens and LNs of wt BALB/c female mice mated with mOVA or wt B6 male mice were incubated with purified chicken OVA rabbit IgG polyclonal Ab and purified FDC-M1 rat IgG Ab, followed by AF488-anti rabbit IgG (1:400) and AF555-anti-rat IgG (1:400), blocked with rat irrelevant IgG, followed by AF647-CD19 Ab.

Cytospins of primary cultures of mouse trophoblast cells were fixed in 4% PF / PBS (15 min, RT), treated with 5% normal goat serum followed by the avidin/biotin blocking kit, and then incubated with (i) biotin-pan-keratin Ab followed by AF488-streptavidin (1:400); (ii) desmin goat IgG polyclonal Ab followed by AF647-anti-goat IgG (1:400).

For detection of CM-DiI-labelled EVs injected i.v. in huMice, tissue cryosections of spleen, bone marrow, thymus, liver and lungs were fixed with 4% PF / PBS (15 min, RT), washed in PBS (5 min, RT) and blocked with 5% goat serum. Cryosections were then incubated with purified anti-human CD163 Ab or anti-human CD169 Ab (both at 1:100) followed by AF488-anti-mouse IgG (1:400). To assess Ab cross-reactivity between human and mouse, human LN and mouse spleen cryosections were incubated with anti-human CD163 (clone

GHI/61) or CD169 (clone 7/2.39) Ab followed by AF488-anti-mouse IgG (1:400), or anti-mouse CD163 (clone S15049I) or CD169 (clone 3D6.112) Ab followed by AF488-anti-rat IgG (1:400).

Primary Ab and secondary reagents were diluted in 5% goat serum, except for the staining with desmin Ab in which horse serum was used. Tissue sections were incubated with the primary Ab overnight at 4°C, and with the secondary reagents for 45 min at RT. Cell nuclei were counterstained with DAPI. Sections were treated with the Vector® TrueVIEW® Autofluorescence Quenching Kit (Vector) and coverslips mounted with Vectashield® vibrance mounting media (Vector), Tissue sections were imaged using a Nikon E800 microscope with Zeiss Axiocam 506 camera.

Immunoprecipitation of mOVA

For immunoprecipitation of OVA from plasma of pregnant BALB/c mice, blood was drawn via cardiac puncture with 3 ml syringes and 27G ½ needles coated with heparin and collected in BD Microtainer® tubes with K_2EDTA (Becton Dickinson). Blood was centrifuged at 2000*g* for 10 min at 4°C, and plasma was collected and diluted to 2 ml final volume with 5mM EDTA / PBS with 1x Halt protease / phosphatase inhibitor cocktail (Thermo Fisher). Next, plasma was centrifuged at 10,000*g* (30 min, 4°C), the supernatant was removed, and the 10,000*g* pellet containing the MVs was resuspended in 500 µl 5mM EDTA / PBS with 1x Halt protease / phosphatase inhibitor cocktail. Five hundred µl of the 10,000*g* supernatant was reserved for the immunoprecipitation, and the remaining supernatant was ultracentrifuged at 100,000*g* to pellet sEVs. After that, the supernatant was collected and the 100,000*g* pellet containing the sEVs was resuspended in 500 µl 5mM EDTA / PBS with 1x Halt protease /phosphatase inhibitor cocktail. Anti-OVA immunoprecipitation was conducted using 50µl of sheep anti-rabbit IgG Dynabeads (Invitrogen) coupled with 2ug of anti-OVA polyclonal rabbit Ab. Five hundred µl of plasma supernatant and EV pellet samples were incubated with 50µl of the Ab-coupled beads (overnight, at 4°C, orbital rotation). After that, the sample was placed on a PureProteome™ magnetic stand (Millipore Sigma), the supernatant was removed, and 100 µl of 0.1M glycine (pH 2.5) were added to the beads to elute the OVA Ag from the beads. The amount of protein in the eluate was measured with a NanoDrop 2000c.

Immunoblotting

For immunoblotting, 6 µg of each sample (resuspended in glycine) was diluted to 50µl with PBS and 4x laemmli sample buffer (BioRad) supplemented with β-mercaptoethanol reducing agent. Samples were heated at 95°C for 10 min and loaded into 10% poly-acrylamide mini-PREOTEAN®TGX™ precast gels (BioRad). Gels were electroblotted on polyvinylidine difluoride membranes (BioRad). For detection of proteins, membranes were probed with a mouse anti-OVA monoclonal Ab diluted 1:500, mouse monoclonal anti-calnexin Ab (1:500), rabbit monoclonal anti-CD63 Ab (1:500), rat monoclonal anti-Grp94 / Hsp90B1 Ab (1:1000), rabbit polyclonal anti-CD81Ab (1:500), rabbit monoclonal anti-GM130 (1:500) and rabbit polyclonal anti-Tsg101 Ab (1:500) in Intercept® Blocking Buffer / 0.1% Tween 20. Membranes were incubated with an IRDye 680-conjugated donkey anti-mouse Igg (Li-Cor) diluted 1:10,000 in in Intercept® Blocking Buffer / 0.1% Tween 20. Membranes were imaged with a Licor Odyssey infrared imaging system.

Isolation of EVs from primary cultures of mouse trophoblasts and mouse plasma

An ExoDiscovery™ Lab Spinner centrifuge fitted with ExoDiscovery™-D20 or -D100 discs were used to isolate by microfluidic tangential flow filtration sEVs from primary mouse trophoblast cultures and pooled plasma of pregnant mice, respectively. Two-day culture supernatants were centrifuged at 2000*g* for 10 min at 4°C, to remove cells and debris. The cleared supernatants were then centrifuged at 10,000*g* for 30 min at 4°C to pellet the MVs, and then filtered through 0.22µm filters (Millipore). The filtered supernatants were then run though the ExoDiscovery™-D20 disc according to the manufacturer's protocol. Final elution volume of the trophoblast sEVs was 600µl in PBS.

For some experiments mouse trophoblast sEVs were lysed by incubation with RIPA buffer (1X, 30 min, RT) followed by sonication on ice water (25 min), and then dialyzed (Slide-A-Lyzer dialysis cassette, MWCO 10,000) against PBS at pH 7 (o/n, 10 ºC).

For flow cytometry analysis of trophoblast sEVs, trophoblast cells from placentas of homozygous $CMV^{Cre/+}$ B6 females impregnated with Exomap1 B6 males were isolated. Trophoblast sEVs were isolated by

ExoDiscovery™ Lab Spinner centrifuge fitted with ExoDiscovery™-D20 disc as described above. Immuno-isolation of Exomap1, *CMV*$^{Cre/+}$ sEVs was performed with streptavidin-Dynabeads (4.5 μm) preincubated with biotin-mouse CD63 Ab (clone NVG-2) and biotin-mouse CD81 Ab (clone Eat-2), or biotin-irrelevant rat IgG2a (clone RTK2758), at a ratio 4 μg of biotin-Ab per 10^7 beads (60 min at room temperature). Beads were washed with PBS and 0.1% bovine serum albumin (BSA) filtered through a 0.2-μm filter, and 25-μg total protein of EVs in 100 μl of PBS and 0.1% BSA was incubated with CD63/CD81 Ab-Dynabeads or irrelevant IgG-Dynabeads (20 μl of beads per sample, from batches of 10^7 beads/ml), in 2 ml Sarstedt tubes, overnight at 4°C, on a sample mixer. Next, tubes were centrifuged to collect the bead-bound EVs in the pellets, and the supernatants (100 μl final volume) were incubated with CD63/CD81 Ab-Dynabeads or irrelevant IgG-Dynabeads (20 μl of beads per sample, from batches of 10^7 beads/ml). After EV incubation, Dynabeads were washed with PBS and 0.1% BSA, transferred to FACS tubes. Beads were analyzed by flow cytometry, and only bead singlets were included in the results.

The amount of protein in the sEV preparations was assessed with a NanoDrop 2000c. The average size of the sEVs was measured with a LM10 NanoSight's instrument equipped with a high-sensitivity electron-multiplying charge-coupled device camera and the NTA 2.0 software (NanoSight), as previously described (1). Samples were measured by NTA at RT, at concentrations ranging from 1.09×10^8 to 10.68×10^8 particles/ml after 1:100 to 1:1000 dilutions in batches of PBS BioPerformance certified for molecular biology (MilliporeSigma) that were previously validated in our LM10 NanoSight's instrument to be particle-free. Samples were run at 12.5 frames/s, with three repeats of 60 s each, within a range of 20 to 60 particles per frame, with more than 350 completed tracks per video, camera shutter speed at 65 ms, pump speed set at 100, and blur and minimum track length set at automatic. NTA parameters were kept identical throughout the whole capture and analysis when comparing different samples. Results were analyzed with the NTA 2.3 software with default settings.

Transmission electron microscopy

Tissue fragments (2 x 2 mm) of spleens from pregnant female mice (E17.5) or harvested on day 7 PP, placentas (E17.5), and cell pellets of primary cultures of mouse trophoblasts obtained by centrifugation (200*g*, 5 min, 4ºC) were fixed in 2% PF - 0.01 % glutaraldehyde – PBS, pH 7.3 (1 hour, on ice). Tissue fragments and cell pellets were transferred to 20% polyvinylpyrrolidone in 1.6 M sucrose buffered with 0.055 M sodium carbonate (overnight, 10 ºC), and then frozen to small stubs in liquid nitrogen. Semi-thin (300 nm) sections were cut on a Leica Ultracut 7 with a cryokit at -110 °C, stained with 0.5% toluidine blue, and examined under a light microscope to determine specific tissue areas. Ultrathin cryosections (65 nm) cut on a Reichart Ultracut and collected on formvar coated copper grids were blocked with 5% normal goat serum – PBS. The grids with the spleen cryosections were labeled with 20 nm gold -conjugated CD169 Ab, 10 nm gold-conjugated CD21/CD35 Ab or a cocktail of 10 nm gold-conjugated CD11c mAbs (clones HL3 and N418) (all primary Abs at 1:100, overnight, 10ºC) in combination with rabbit polyclonal OVA Ab (1:500, overnight, 10ºC) and 6nm gold goat anti-rabbit IgG Ab (1:50, 1 hour, RT). The grids with the mouse trophoblast cell pellets and placenta cryosections were labeled with rabbit polyclonal OVA Ab (1:500, overnight, 10ºC) and then incubated with 6nm gold goat anti-rabbit IgG Ab (1:50, 1 hour, RT). Following staining, grids were rinsed in PBS, sections were fixed in 2.5% glutaraldehyde and then rinsed in PBS and distilled water. The rinsed sections were counterstained with 2% neutral uranyl acetate and 4% uranyl acetate, and then coated with methyl cellulose. All specimens were analyzed on a JEOL 1400 transmission electron microscope with a side mount AMT 2k digital camera.

Adoptive transfer of TCR transgenic T cells

CD8 T cells were purified from spleens and lymph nodes of CD90.1 Rag1$^{-/-}$ OT-I B6 mice using a CD8 Dynabead negative isolation kit, stained with 7.5 µM Vibrant CFDA SE Cell Tracer (Invitrogen), and administered i.v. at 3×10^6 T cells per mouse. Mice were left untreated, injected i.p with soluble OVA (100 µg / mouse) plus agonistic CD40 Ab (FGK45.5, 150 µg per mouse) and poly I:C (10 µg / mouse), or injected i.v. with 100 µg of sEVs purified from primary cultures of mOVA B6 male x wt BALB/c, or control wt B6 male x wt BALB/c female mice trophoblast cell culture supernatants.

Flow cytometry analysis

Analysis of the T cell response against mOVA delivered via trophoblast sEVs was done on spleens from wt B6 female mice previously i.v. injected with CFSE-labelled OT-I T cells, and then left untreated, i.p. injected with soluble OVA + CD40 Ab + poly I:C, or i.v. treated with the trophoblast sEVs. Spleens were teased apart with fine forceps, filtered through 70 µm cell strainers, and spleen erythrocytes were depleted with NH_4Cl lysis buffer. Splenocytes were incubated with CD16/CD32 Ab to block FcR, and then labelled (30 min, 4ºC) with: (i) BUV395-CD3 Ab, BV605-CD8 Ab, APC-CD90.1 Ab, PerCP-Cy5.5-CD69 Ab, PE-Cy7-CD62L Ab, BV421-TCRV2 Ab and eF780-Fixable Viability Dye (FVD) or (ii) BUV395-CD3 Ab, BV605-CD8 Ab, APC-CD90.1 Ab, BUV395-CD3 Ab, BV605-CD8 Ab, APC-CD90.1 Ab, PerCP-Cy5.5-CD366 Ab, PE-Cy7-CD279, and BUV-737-CD223 and NucView® 405 Caspase 3 substrate (1:1000, Biotium). After labelling, cells were fixed in 4% PF / PBS.

For intracellular staining, splenocytes were plated in 6 well plates ($2x10^7$ cells per well) and incubated with eBioscience™ Cell Stimulation Cocktail (18 hours, 37 °C). Cells were harvested and surface labeled with BUV395-CD3 Ab, BV605-CD8 Ab and APC-CD90.1 Ab (30 min, 4°C). Next, cells were fixed with 4% PFA / PBS (20 min, at RT), permeabilized with 0.1% saponin / 1% FBS / PBS solution and labelled with PerCP-Cy5.5-IFN-γ Ab alone or in combination with Pacific blue-GranzymeB Ab (30 min, at 4°C). Cells were washed with permeabilization buffer, centrifuged, and resuspended in staining buffer prior to analysis. The OT-I CD8 T response was analyzed by FACS, 2 days after i.v. injection of the sEVs.

For flow cytometry assessment of human leukocyte chimerism in huMice, peripheral blood was collected in BD Microtainer tubes with K_2EDTA (Becton Dickinson) and erythrocytes were lysed with Red Blood Cell Lysing Buffer Hybri-Max™ (Sigma) (15 min, RT). Peripheral leukocytes were then incubated with anti-human CD16/32 Ab and anti-mouse CD16/32 Ab to block mouse and human FcR, following by incubation with PE-Cy7 anti-mouse CD45 and BUV737 anti-human CD45. For evaluation of human leukocyte chimerism in spleen, splenocytes of huMice were treated with Red Blood Cell Lysing Buffer Hybri-Max™ (15 min, RT), rinsed in

PBS, incubated with anti-human CD16/32 Ab and anti-mouse CD16/32 Ab. Next, splenocytes were incubated with the following combination of Abs: PE-Cy7 anti-mouse CD45, BUV737 anti-human CD45, FITC anti-human CD3, APC-Cy7 anti-human CD4, BV421 anti-human CD8 and APC anti-human CD19; or PE anti-human CD45 and BV605 anti-human CD33.

In flow cytometry analysis fluorochrome-conjugated Ab were used between 1:100 to 1:200 final concentration, diluted in PBS / 1% FBS / 0.1 % sodium azide (pH 7.4-7.6). In all experiments, appropriate irrelevant Ab were used as controls, and cells were fixed in 4 % PF / PBS. Data were acquired with a BD LSR II flow cytometer and analyzed using FlowJo v10 software.

High resolution liquid chromatography - mass spectrometry

The samples were thawed at RT, vortexed (10 min), sonicated (5 min, water bath) and centrifuged (10 min, 13,000*g*, RT). Total protein was quantified by micro-BCA assay (Thermo Scientific) and 10 µg of protein from each sample were digested according to the S-trap (Protifi) manufacturer protocol. Digested samples were subject to reduction with 20 mM dithiothreitol (95°C, 10 min), cooled to RT, and alkylated by incubation with 40 mM iodoacetamide (in darkness, 30 min) followed by centrifugation (8 min, 13,000*g*), acidified with 12% phosphoric acid at 1:10 concentration, and diluted six-fold in binding buffer containing 90% methanol and 100 mM tetraethylammonium bromide (TEAB) at pH 7.1. Samples were then dispensed onto S-trap columns that were washed with binding buffer and centrifuged at (1m, 4,000*g*). Columns were washed with binding buffer, centrifuged (1 min, 4,000*g*), and transferred to clean tubes for incubation at 47°C in 50 mM TEAB for Lys-C digestion overnight followed by trypsin digestion for 1 h (1:20 enzyme/substrate). Samples were eluted with a series of solutions including 50 mM TEAB, 0.2% formic acid, and 50% acetonitrile with 0.2% formic acid, and underwent centrifugation (1 min, 1000*g*) for each eluent added. Samples were then dried in a speedvac and resuspended in a solution of 3% acetonitrile and 0.1% trifluoroacetic acid and desalted using Pierce Peptide Desalting Spin Columns (Thermo Scientific). Eluants were dried in a speedvac and resuspended in a solution of 3% acetonitrile and 0.1% formic acid to a final concentration of 0.2 µg/µL.

LC-MS analysis was conducted on a Bruker timsTOF Pro2 coupled to a NanoElute 2. Approximately 200 ng of peptide was loaded onto an Aurora Ultimate C18 column (1.7 µm, 25 cm x 75 µm, IonOpticks) and eluted at 300 nl/min over a 60-minute gradient. The timsTOF Pro2 was set to PASEF scan mode and DDA with a scan range of 100 - 1700 m/z with 10 PASEF ramps. The TIMS was set to a 100 ms ramp and accumulation time (100% duty cycle) with a ramp rate of 9.43 Hz. Linear precursor repetitions were set to a target intensity of 20,000 and a target threshold of 2500 and active exclusion was set to 0.40 min. Collision energy was set to a base of 1.60 $1/K_0$ [V-s/cm^2] at 59 eV and 0.60 $1/K_0$ [V-s/cm^2] at 20 eV. An isolation width was set to 2 m/z for <700 m/z and 3 m/z for >800 m/z.

Mass spectrometry data analysis

Initial MS data analysis was carried out in MaxQuant v2.6.5.0. MS/MS spectra were searched against the mouse SwissProt database using Andromeda set to default Bruker TIMS settings.

A Student's t-test on median normalized intensity values was used to determine the statistical significance of the difference between the trophoblast cell and trophoblast sEV samples. Proteins with a single-peptide identification were excluded from the data analysis. The correction of statistical test results was performed by the Benjamini–Hochberg false discovery rate (FDR). A protein was considered as a significant protein if the FDR was ≤ 0.05.

Human placentas and dispersed primary human trophoblasts

All placentas used in our studies were obtained from uncomplicated pregnancies and term deliveries at Magee-Womens Hospital in Pittsburgh, under a protocol that was approved by the Institutional Review Board at the University of Pittsburgh. PHT cells were isolated using the trypsin-DNase-dispase/Percollx method as described by Kliman et al. (1986) (2), with modifications as we previously published (3, 4).

Generation and purification of EVs from primary human trophoblasts

EVs were purified from 72-hour culture supernatants of PHT cells cultured in DMEM containing 10% EV-free bovine growth serum, and penicillin / streptomycin at 37◦ C in a 5% CO_2 air atmosphere. Supernatants were centrifuged at 2000*g* (20 min, 4°C) followed by passage through a 0.22µm filter and then centrifuged 10,000*g* (30 min, 4°C). The 10,000*g* supernatants were concentrated by ultrafiltration on sterile Vivacell 100 (Sartorius) filters (2800*g*, 35 min, 4°C). The filtered supernatants were adjusted to a volume of 11 ml with PBS and centrifuged at 110,000*g* for 16h at 4°C. The 110,000*g* pellets with the EVs were incubated with CM-DiI–PBS (0.1 µg/µl, 300 µl final volume), at 37°C for 5 min, and then on ice for 20 min. The CM-DiI–labeled EVs were purified from the free dye using iodixanol (OptiPrep) gradients, as previously described (5), with minor modifications. Briefly, 300 µl of CM-DiI– labeled EVs were mixed with 6 ml of 30% (w/v) OptiPrep solution in sucrose buffer [0.25 M sucrose, 10 mM tris (pH 8.0), and 1 mM EDTA, pH 7.4], allowed 30-min mixing on a rocker to equilibrate the solution, and then transferred into Ultra-Clear (Beckman Coulter) sterile 11-ml tubes. Next, 3 ml of 20% (w/v) OptiPrep solution in sucrose buffer, followed by 2.5 ml of 10% (w/v) OptiPrep solution in sucrose buffer, was successively layered on top on the 30% OptiPrep solution containing the EVs. The tubes with the OptiPrep gradients were centrifuged at 110,000*g* (16 hours, 4°C). The fractions containing the EVs were harvested, washed in PBS, and centrifuged at 110,000*g* for 90 min, and the EV pellets were collected in 300 µl of PBS. The amount of protein in the EV preparations was assessed with a NanoDrop 2000c. The average size of the EVs was measured with a LM10 NanoSight's instrument as described above.

Amnis ImageStream analysis

Single cell suspensions of splenocytes from huMice i.v. injected with CM-DiI-labeled human PHT-derived sEVs, were FcR– blocked with anti-human and anti-mouse CD16/CD32 Abs and incubated (30 min at 4°C) with the following Ab: FITC-human CD1c, PerCP-humanCD19, Pacific Blue-HLA-DR, BV605-human CD45, APC-human CD141, and APC-Cy7-human CD3. Fluorochrome-conjugated Abs were used between 1:100 and 1:200 final concentration, diluted in PBS/1% FBS/0.1% sodium azide solution (pH 7.4 to 7.6). Cells were fixed in 4% PF-PBS immediately after labeling. Cells (2×10^5 per group) were analyzed with a two-laser Amnis ImageStream analyzer at a magnification of ×60. Cell images were analyzed with the software IDEAS v6.2.

Events in focus were gated by plotting the gradient root mean square (RMS) feature of the bright field of camera 1 (channel 1) against the gradient RMS feature of the bright field of camera 2 (channel 9). From the events in focus, single cells were identified as events with low area and high aspect ratio intensity (bright field of camera 1, channel 1). Single cells were selected for further analysis based on the following phenotypes: $CD45^{Pos}$ $CD3^{Pos}$ $CD19^{Neg}$ (human T cells), $CD45^{Pos}$ $CD3^{Neg}$ $CD19^{Pos}$ (human B cells), $CD45^{Pos}$ $CD3^{Neg}$ $CD19^{Neg}$ HLA-DR^{Pos} $CD141^{Pos}$ $CD1d^{Neg}$ (human cDC1) and $CD45^{Pos}$ $CD3^{Neg}$ $CD19^{Neg}$ HLA-DR^{Pos} $CD141^{Neg}$ $CD1c^{Pos}$ (human cDC2).

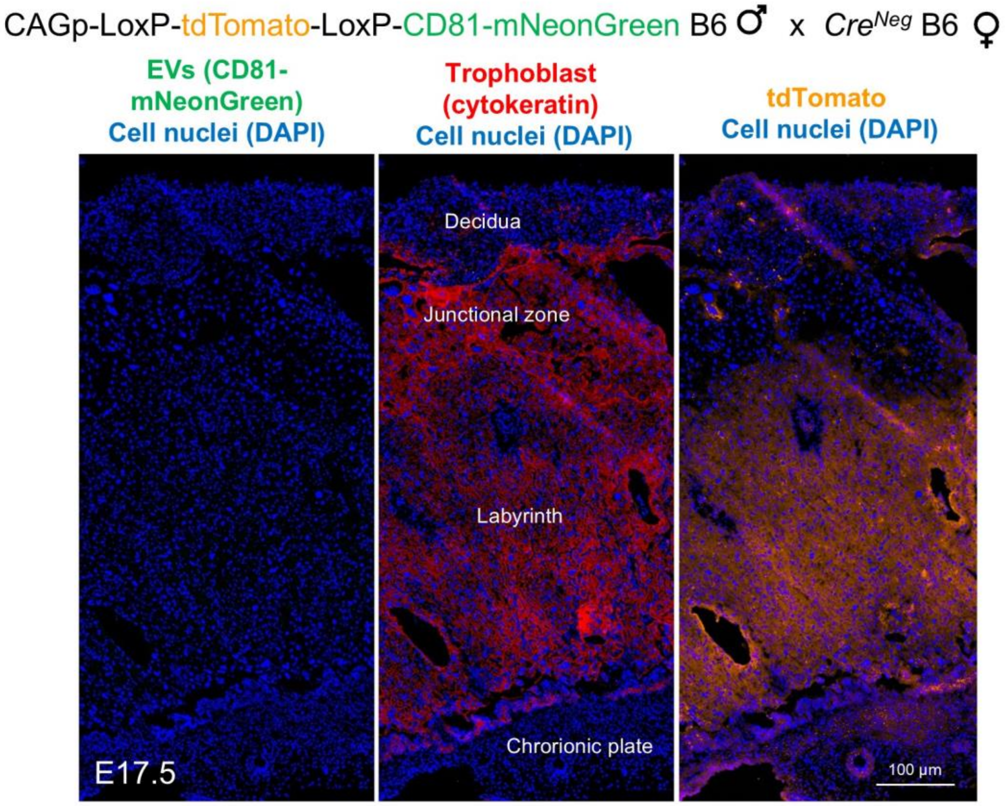

Figure S1. Expression of tdTomato in control mouse trophoblast. As a negative control, detection by fluorescence microscopy of tdTomato and absence of CD81-mNeonGreen on a cryosection of a placenta (E17.5) from a *Cre*^{Neg} B6 female impregnated by a Exomap1 B6 male. Magnification, x200.

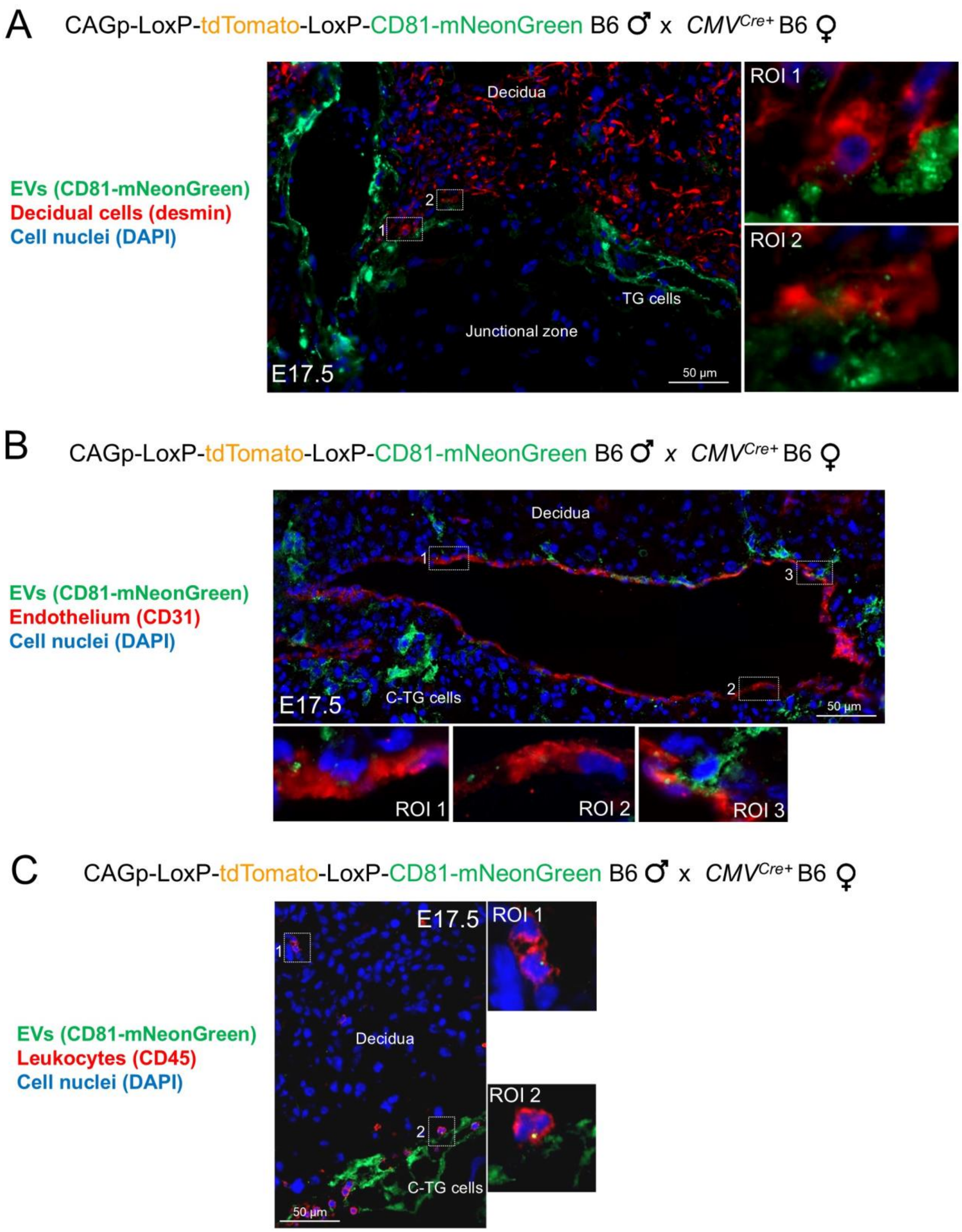

Figure S2. **Feto-placental sEVs at the maternal-fetal interface. A)** Detection by immunofluorescence microscopy on cryosections, of CD81-mNeonGreen as a punctate pattern, attached to or within decidual stromal (desmin^{Pos}) cells adjacent to canal-associated trophoblast giant cells. ROI: region of interest. **B)** CD81-mNeonGreen^{Pos} content in endothelial (CD31^{Pos}) cells of maternal blood vessels in the decidua in proximity to CD81-mNeonGreen^{Pos} trophoblasts. **C)** CD81-mNeonGreen^{Pos} content in decidua-infiltrating leukocytes (CD45^{Pos} cells). In (A-C), images are representative of 8 placentas from 3 pregnant females. Magnification, x200.

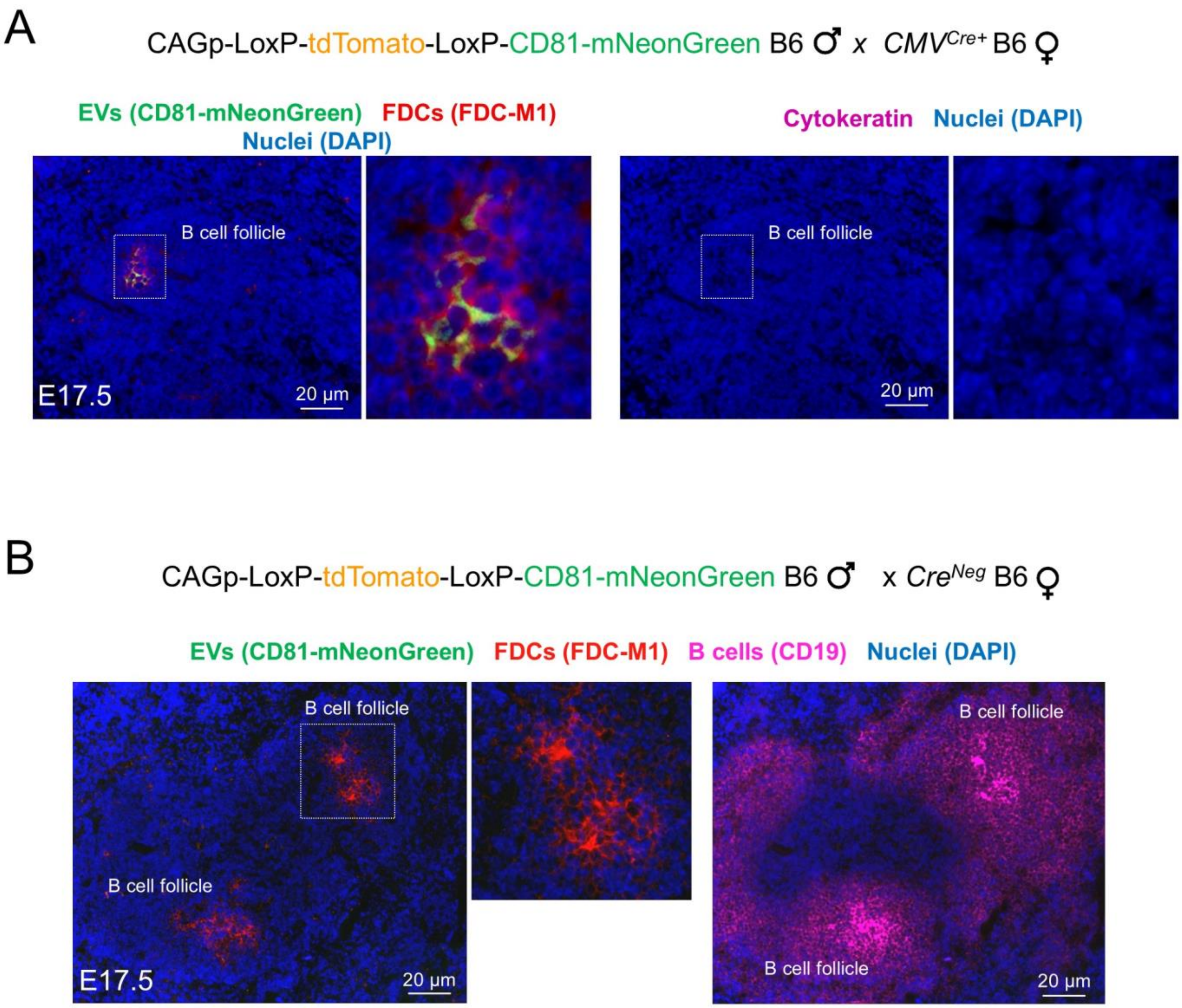

Figure S3. Detection of fetoplacental-derived CD81-mNeonGreen in the maternal spleen is not caused by trophoblast-derived tissue fragments. A) Immunofluorescence microscopy of a cryosection of the spleen of a *CMV*$^{Cre+}$ B6 female impregnated by a Exomap1 B6 male did not contain detectable cytokeratin^{Pos} trophoblast-derived fragments. Magnification, x200. **B)** CD81-mNeonGreen^{Pos} material was undetectable in spleens of *Cre*^{Neg} B6 females impregnated with homozygous Exomap1 B6 males. In (A) and (B), images are representative of 3-4 spleens analyzed. Magnification, x200.

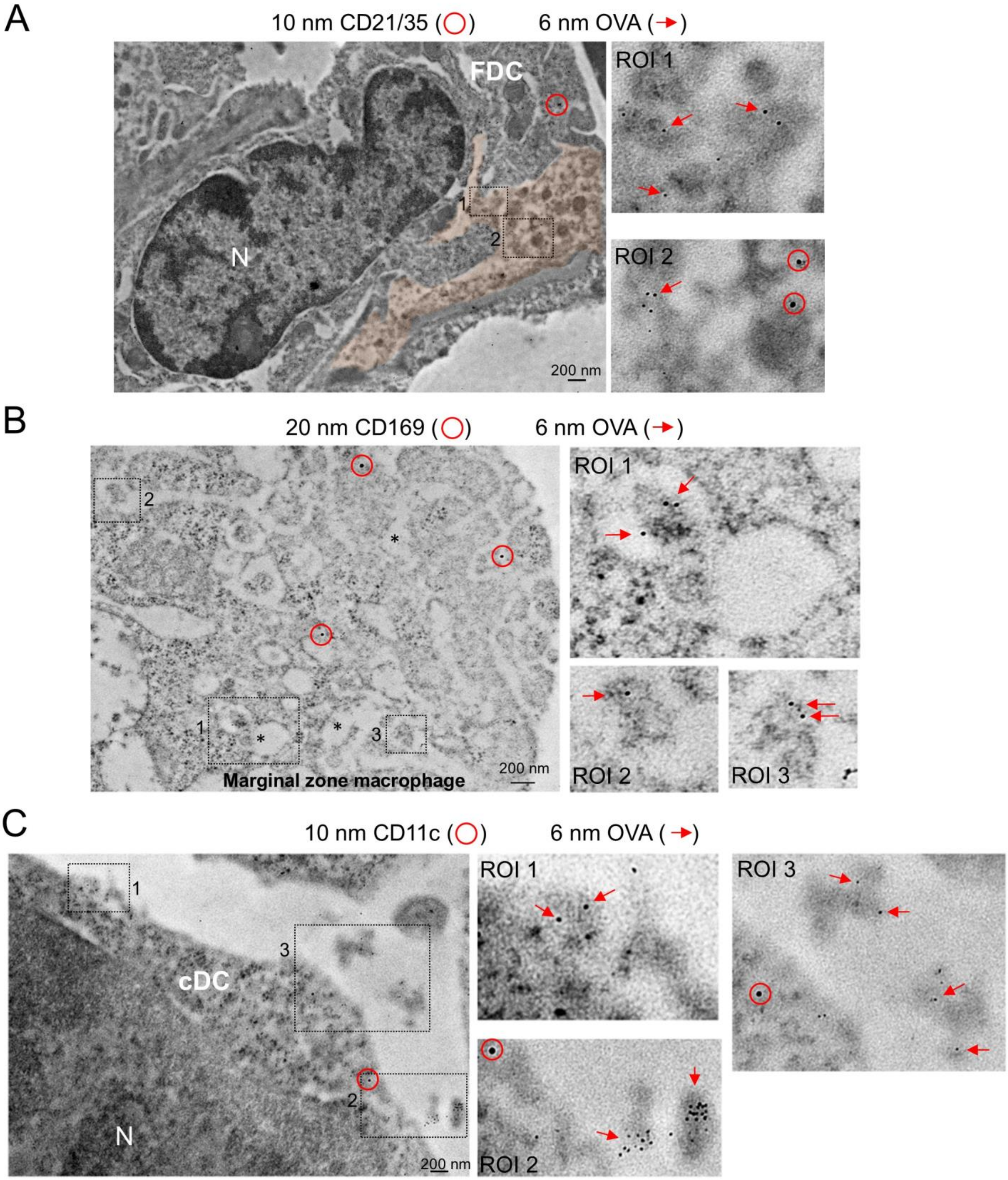

Figure S4. Detection of sEVs bearing fetoplacental Ag in maternal immune cells in the spleen. A-C) IEM analysis on ultrathin cryosections of spleens of BALB/c females impregnated with mOVA B6 males analyzed on E17.5. Cryosections were labelled with 6 nm OVA Ab in combination with 10 nm CD21/35 Ab (A), 20 nm CD169 Ab (B) or 10 nm CD11c Ab (C) to identify FDC, MZ macrophages and cDCs, respectively. Magnifications X20,000-80,000.

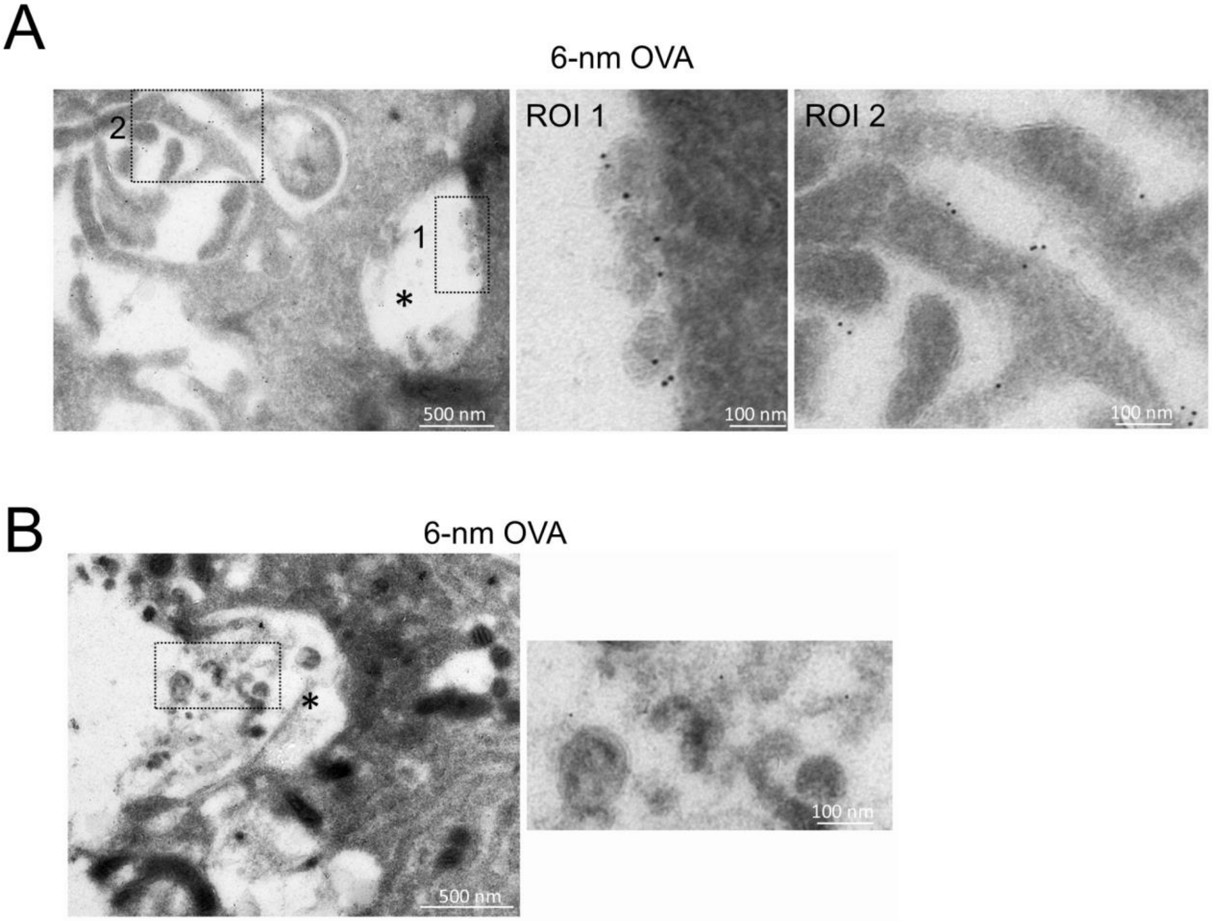

Figure S5. Ultrastructural analysis of localization of mOVA on mouse trophoblast cells. A) Detection of mOVA by IEM on ultrathin cryosections on sEVs inside a multivesicular body (asterisk, ROI 1) or on the surface (ROI 2) of a trophoblast cell from a primary culture of trophoblasts from placentas of BALB/c female mice impregnated with mOVA B6 males (E12.5 - E17.5). **B)** IEM of cells from primary culture of trophoblasts from placentas of BALB/c female mice impregnated with mOVA B6 males (E12.5 - E17.5) showing a trophoblast cell discharging its content of intraluminal vesicles, some of them carrying mOVA (inset), from a multivesicular body (asterisk) to the extracellular milieu. In (A) and (B) images are representative of 3 different samples. Magnification, x40,000. ROI: region of interest.

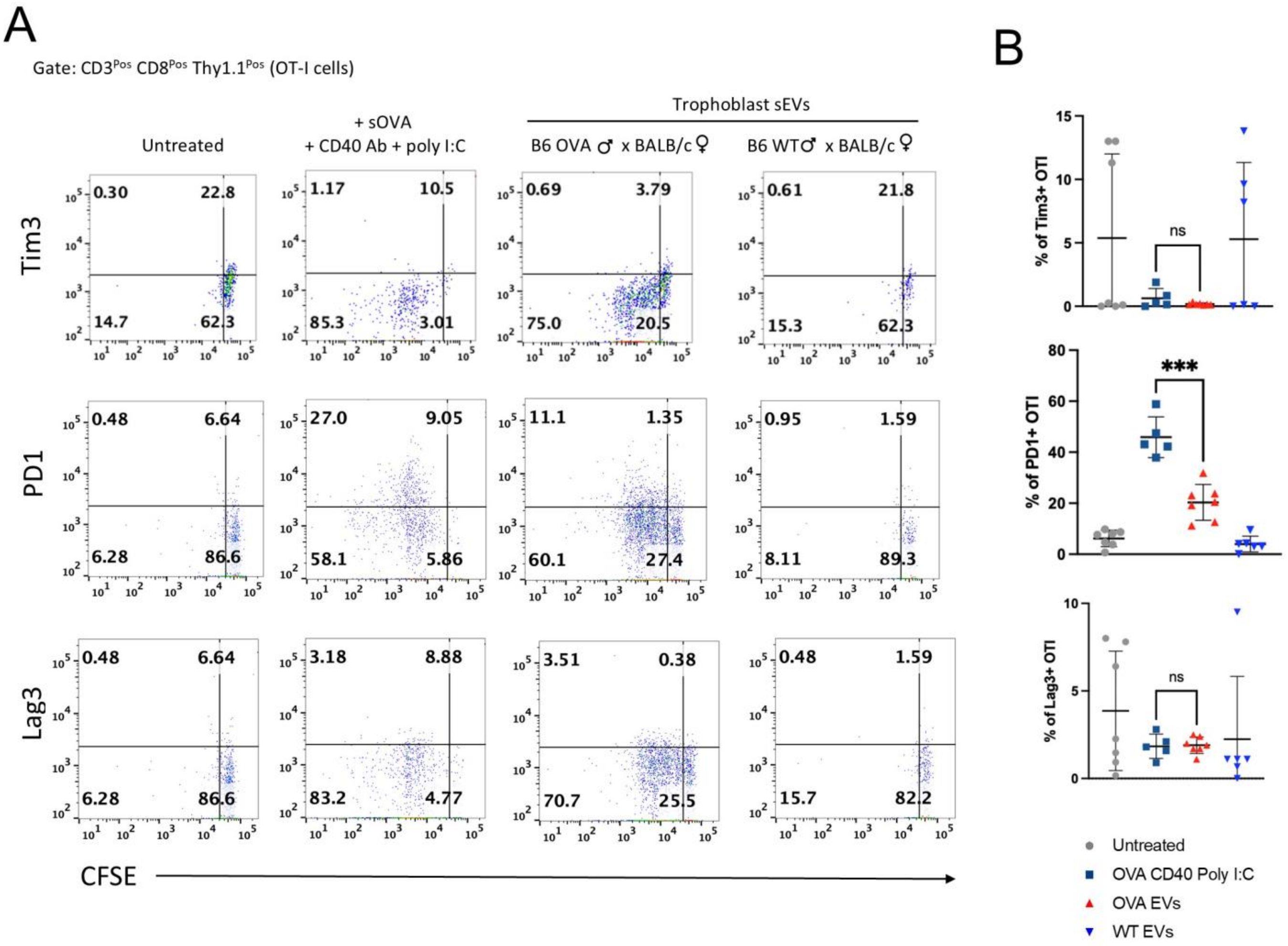

Figure S6. **I.v. injection of trophoblast sEVs and T cell exhaustion in the spleen. A)** Representative flow cytometric analysis of splenocytes of B6 virgin females (CD90.2) i.v. infused with CFSE-labelled OT-I CD8 T cells (CD90.1) and injected 24 hours later with sEVs isolated from primary trophoblast culture supernatants from BALB/c females mated with mOVA B6 males or control wt B6 males. As a positive control of OT-I cell activation, a group was injected i.p. with soluble OVA + agonistic CD40 Ab + poly I:C, 24 hours after the OT-I cell transfer. Splenocytes were analyzed by FACS, 2 days after sEV administration. **B)** Percentages of $Tim3^{+}$, $PD1^{+}$ and $Lag3^{+}$ OT-I cells in the spleens from the experiment shown in (A). Each dot represents one mouse. Error bars: means ± SD. *** $P < 0.001$. ns: not significant.

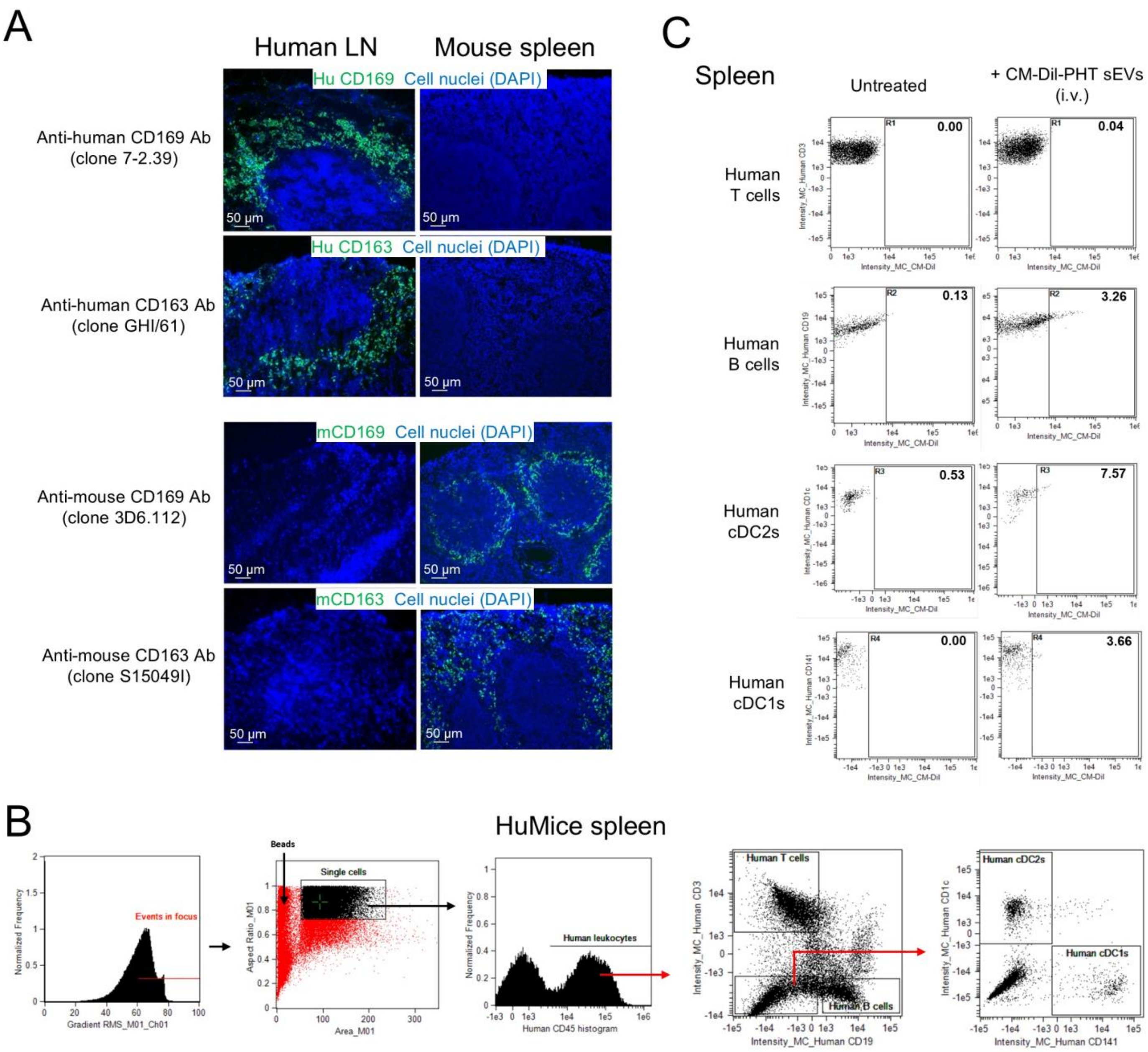

Figure S7. Primary human trophoblast (PHT) sEVs traffic to huMice tissues. A) Analysis by fluorescence microscopy on sections of human lymph nodes (LN) and mouse spleens of species cross-reactivity of the Abs against human CD169 and CD163 used in the huMice experiments. **B)** Cell gating strategy used for ImageStream analysis of splenocytes of huMice left untreated or injected i.v. with CM-Dil-PHT sEVs. **C)** Dot plots with ImageStream analysis of CM-DiI associated to human T cells, B cells, and cDCs in splenocytes of huMice untreated or injected i.v. with CM-DiI-PHT sEVs. Representative of 4-6 huMice in the experimental group and 2 huMice in the untreated group. ImageStream analysis was done 18 hours after injection of CM-DiI-PHT sEVs.

Table S1. sEV-associated proteins enriched in trophoblast sEVs compared to trophoblast cells.

**sEVs phenotype, enriched protein family and function**	**Proteins**
ESCRT	Vps4b, Vps26b, Vps35 and Vta1.
Traffic and sorting, and ESCRT accessory	Flotillin, TSG101, Syntenin-1 and Alix.
Tetraspanins	Tspan8, Tspan14, Tspan31, CD9, CD63, CD81,CD82, and CD151.
Rab family	Rab1a/b, Rab2a, Rab3gap2, Rab3d, Rab4a/b, Rab5b, Rab5a, Rab6a, Rap1a/b, Rab7a, Rab8a/b, Rab10, Rab11a/b, Rab14, Rab18, Rab21, Rab22a, Rab32, Rab24 and Rab35.
SNARE's (STX)	Stx7, Stxbp3, Stx7, Vamp8, Stxbp1, Stxbp2, Stxbp5.
Heat shock proteins and chaperons	Hsp90aa1, Hsp90ab1, Hmga1, Hmgb1, Hmgn1 and Hmgn2.

Table S2. Proteins detected by LC-MS with anti-inflammatory or immunosuppressive effects significantly enriched in trophoblast sEVs compared with their trophoblast parent cells.

UniProt accession	Protein name (symbol)	Log_2 ↑ FC	$-Log_{10}$ (*p* value)	Anti-inflammatory / immunosuppressive effect	References
P97298	Pigment epithelium-derived factor (PEDF)	11.83	6.2	Anti-inflammatory. Reduces apoptosis and LPS-induced inflammation of decidual stromal cells. Suppresses DC maturation and promotes the function of Tregs cells.	(6-8)
Q9Z1R3	Apolipoprotein M (ApoM)	10.47	3.23	Anti-inflammatory. Reduces monocyte secretion of IL-6, IL-1β and TNF-α.	(9)
P08226	Apolipoprotein E (Apo E)	8.10	4.14	Anti-inflammatory and immunosuppressive. Inhibits secretion of IL-6, IL-1β and TNF-α, while inducing IL-10 secretion by DCs. Promotes T cell arrest and apoptosis.	(10, 11)
Q03350	Thrombospondin 2 (TSP2)	7.19	3.09	Anti-inflammatory and immunosuppressive. Inhibits the pro-inflammatory effect of LPS and promotes the secretion of IL-10 and expression of IL-10R in Treg cells.	(12, 13)
O35375	Neuropilin-1 (NRP1)	6.93	4.21	Anti-inflammatory and immunosuppressive. Maintains Treg stability and immunosuppressive function. Facilitates APC-T cell trogocytosis that sensitizes CD4 T cells to the inhibitory signaling of Sema3A.	(14, 15)
P32261	Antithrombin III (ATIII)	6.78	4.17	Anti-inflammatory, immunosuppressive and antimicrobial. Prevents activation of NF-kβ by binding the inflammatory inhibitor receptors LRP-1, CD13 and CD300f. Inhibits IL-2, IL-4 and IFN-γ transcripts and cell proliferation in T cells, and B cell activation.	(16, 17)
P35441	Thrombospondin 1 (TSP1)	6.75	4.49	Anti-inflammatory. Prevents interaction between CD14 and CD47 in macrophages and inhibits NF-kβ/AP1 signaling and secretion of IL-1β, activates TGFβ1, and downregulates nitric oxide signaling.	(13, 18, 19)
Q64449	Mannose receptor (MRC2)	6.18	4.64	Anti-inflammatory and immunosuppressive. Induces DCs to secret anti-inflammatory mediators such as IL-1R antagonist, IL-1 type II and IL-10 without inducing the secretion of IL-12. Prevents Th1 polarization and amplifies the generation of Treg cells.	(20, 21)
Q9DAS4	Prolactin 8A8 (PRL-8A8)	4.24	3.13	Anti-inflammatory. Reduces the LPS-TLR4 activation of NF-kβ resulting in decreased secretion of IL-1β, TNF-α and IL-6.	(22)
O70435	Laminin A1 (LAMA1)	4.15	2.85	Anti-inflammatory. The LAMA1 peptide IKVAV, prevents type 1 macrophage (M1) bias. LAMA1 also reduces secretion of IL-1β, IL-6, IL12 and IFN-γ in a model of colitis.	(23, 24)
Q9CQ58	Prolactin 7A2 (PRL-7A2)	3.85	2.75	Anti-inflammatory. Reduces LPS-TLR4 activation of NF-kβ resulting in decreased secretion of IL-1β, TNF-α and IL-6.	(22)
Q9CQ58	Prolactin 8A9, (PRL 8A9)	3.02	2.81	Anti-inflammatory. Dampens LPS-TLR4 activation of NF-kβ resulting in decreased secretion of IL-1β, TNF-α and IL-6.	(22)
Q3UKK2	Carcinoembryonic antigen-related cell adhesion molecule 5 (CEACAM5)	2.21	4.23	Immunosuppressive. Promotes suppressive CD8 T cell cells and reduces NK function.	(25, 26)
P09535	Insulin like growth factor receptor 2 (IGF2R)	1.49	2.8	Anti-inflammatory and immunosuppressive. Promotes differentiation of anti-inflammatory macrophages, activates TGFβ, inhibits T cell proliferation and induces IL-10 secretion by B cells	(27-29)
Q07797	Galectin-3-binding protein (Gal-3BP)	1.32	3.6	Immunosuppressive. Prevents DC maturation and activation of CD8 T cells	(30)

References for Supplemental Methods and Supplemental Tables S1 and S2.

1. Gardiner C, Ferreira YJ, Dragovic RA, Redman CW, and Sargent IL. Extracellular vesicle sizing and enumeration by nanoparticle tracking analysis. *J Extracell Vesicles.* 2013;2.
2. Kliman HJ, Nestler JE, Sermasi E, Sanger JM, and Strauss JF, 3rd. Purification, characterization, and in vitro differentiation of cytotrophoblasts from human term placentae. *Endocrinology.* 1986;118(4):1567-82.
3. Nelson DM, Johnson RD, Smith SD, Anteby EY, and Sadovsky Y. Hypoxia limits differentiation and up-regulates expression and activity of prostaglandin H synthase 2 in cultured trophoblast from term human placenta. *Am J Obstet Gynecol.* 1999;180(4):896-902.
4. Mouillet JF, Chu T, Nelson DM, Mishima T, and Sadovsky Y. MiR-205 silences MED1 in hypoxic primary human trophoblasts. *FASEB J.* 2010;24(6):2030-9.
5. Kowal J, Arras G, Colombo M, Jouve M, Morath JP, Primdal-Bengtson B, et al. Proteomic comparison defines novel markers to characterize heterogeneous populations of extracellular vesicle subtypes. *Proc Natl Acad Sci U S A.* 2016;113(8):E968-77.
6. Zheng J, Li Y, Sang Y, Xu L, Jin X, Tao Y, et al. Pigment epithelium-derived factor, a novel decidual natural killer cells-derived factor, protects decidual stromal cells via anti-inflammation and anti-apoptosis in early pregnancy. *Hum Reprod.* 2020;35(7):1537-52.
7. Singh RB, Blanco T, Mittal SK, Taketani Y, Chauhan SK, Chen Y, et al. Pigment Epithelium-derived Factor secreted by corneal epithelial cells regulates dendritic cell maturation in dry eye disease. *Ocul Surf.* 2020;18(3):460-9.
8. Singh RB, Blanco T, Mittal SK, Alemi H, Chauhan SK, Chen Y, et al. Pigment Epithelium-Derived Factor Enhances the Suppressive Phenotype of Regulatory T Cells in a Murine Model of Dry Eye Disease. *Am J Pathol.* 2021;191(4):720-9.
9. Yao S, Luo G, Liu H, Zhang J, Zhan Y, Xu N, et al. Apolipoprotein M promotes the anti-inflammatory effect of high-density lipoprotein by binding to scavenger receptor BI. *Ann Transl Med.* 2020;8(24):1676.
10. Wu X, Srinivasan P, Basu M, Zhang P, Saruwatari M, Thommandru B, et al. Tumor Apolipoprotein E is a key checkpoint blocking anti-tumor immunity in mouse melanoma. *Front Immunol.* 2022;13:991790.
11. Zhang HL, Wu J, and Zhu J. The immune-modulatory role of apolipoprotein E with emphasis on multiple sclerosis and experimental autoimmune encephalomyelitis. *Clin Dev Immunol.* 2010;2010:186813.
12. Papageorgiou AP, Swinnen M, Vanhoutte D, VandenDriessche T, Chuah M, Lindner D, et al. Thrombospondin-2 prevents cardiac injury and dysfunction in viral myocarditis through the activation of regulatory T-cells. *Cardiovasc Res.* 2012;94(1):115-24.
13. Li Q, Fu X, Yuan J, and Han S. Contribution of Thrombospondin-1 and -2 to Lipopolysaccharide-Induced Acute Respiratory Distress Syndrome. *Mediators Inflamm.* 2021;2021:8876484.
14. Chuckran CA, Liu C, Bruno TC, Workman CJ, and Vignali DA. Neuropilin-1: a checkpoint target with unique implications for cancer immunology and immunotherapy. *J Immunother Cancer.* 2020;8(2).
15. Roy S, Bag AK, Singh RK, Talmadge JE, Batra SK, and Datta K. Multifaceted Role of Neuropilins in the Immune System: Potential Targets for Immunotherapy. *Front Immunol.* 2017;8:1228.
16. Zuo XJ, Nicolaidou E, Okada Y, Toyoda M, and Jordan SC. Antithrombin III inhibits lymphocyte proliferation, immunoglobulin production and mRNA expression of lymphocyte growth factors (IL-2, gamma-IFN and IL-4) in vitro. *Transpl Immunol.* 2001;9(1):1-6.
17. Schlommer C, Brandtner A, and Bachler M. Antithrombin and Its Role in Host Defense and Inflammation. *Int J Mol Sci.* 2021;22(8).

18. Stein EV, Miller TW, Ivins-O'Keefe K, Kaur S, and Roberts DD. Secreted Thrombospondin-1 Regulates Macrophage Interleukin-1beta Production and Activation through CD47. *Sci Rep.* 2016;6:19684.
19. Kaur S, and Roberts DD. Emerging functions of thrombospondin-1 in immunity. *Semin Cell Dev Biol.* 2024;155(Pt B):22-31.
20. Chieppa M, Bianchi G, Doni A, Del Prete A, Sironi M, Laskarin G, et al. Cross-linking of the mannose receptor on monocyte-derived dendritic cells activates an anti-inflammatory immunosuppressive program. *J Immunol.* 2003;171(9):4552-60.
21. Redelinghuys P, and Brown GD. Inhibitory C-type lectin receptors in myeloid cells. *Immunol Lett.* 2011;136(1):1-12.
22. Flores-Espinosa P, Mendez I, Irles C, Olmos-Ortiz A, Helguera-Repetto C, Mancilla-Herrera I, et al. Immunomodulatory role of decidual prolactin on the human fetal membranes and placenta. *Front Immunol.* 2023;14:1212736.
23. Jha A, and Moore E. Laminin-derived peptide, IKVAV, modulates macrophage phenotype through integrin mediation. *Matrix Biol Plus.* 2024;22:100143.
24. Spenle C, Lefebvre O, Lacroute J, Mechine-Neuville A, Barreau F, Blottiere HM, et al. The laminin response in inflammatory bowel disease: protection or malignancy? *PLoS One.* 2014;9(10):e111336.
25. Roda G, Jianyu X, Park MS, DeMarte L, Hovhannisyan Z, Couri R, et al. Characterizing CEACAM5 interaction with CD8alpha and CD1d in intestinal homeostasis. *Mucosal Immunol.* 2014;7(3):615-24.
26. Shi H, Tsang Y, and Yang Y. Identification of CEACAM5 as a stemness-related inhibitory immune checkpoint in pancreatic cancer. *BMC Cancer.* 2022;22(1):1291.
27. Wang X, Lin L, Lan B, Wang Y, Du L, Chen X, et al. IGF2R-initiated proton rechanneling dictates an anti-inflammatory property in macrophages. *Sci Adv.* 2020;6(48).
28. Song D, Wu Y, Li J, Liu J, Yi Z, Wang X, et al. Insulin-like growth factor 2 drives fibroblast-mediated tumor immunoevasion and confers resistance to immunotherapy. *J Clin Invest.* 2024;134(22).
29. Geng XR, Yang G, Li M, Song JP, Liu ZQ, Qiu S, et al. Insulin-like growth factor-2 enhances functions of antigen (Ag)-specific regulatory B cells. *J Biol Chem.* 2014;289(25):17941-50.
30. Kouo T, Huang L, Pucsek AB, Cao M, Solt S, Armstrong T, et al. Galectin-3 Shapes Antitumor Immune Responses by Suppressing CD8+ T Cells via LAG-3 and Inhibiting Expansion of Plasmacytoid Dendritic Cells. *Cancer Immunol Res.* 2015;3(4):412-23.

Table S3. **Resources**

REAGENT or RESOURCE	SOURCE	IDENTIFIER
Antibodies		
Anti-Mouse CD3 (Clone 17A2), purified	eBioscience	Cat # 14-0032-85
Anti-Mouse CD3 (Clone 17A2), BUV395-conjugated	BD	Cat #569614
Anti-Mouse CD4 (Clone RM4-5), BV605-conjugated	Biolegend	Cat # 100548
Anti-Mouse CD8a (Clone 53-6.7), BV605-conjugated	BD	Cat # 563152
Anti-Mouse CD11c (Clone HL3), purified	BD Pharmingen	Cat# 553799
Anti-Mouse CD11c (Clone N418), purified	BioLegend	Cat# 117302
Anti-Mouse CD16/CD32 (Clone 93), purified	Thermo Fisher	Cat # 14-0161-86
Anti-Mouse CD19 (Clone 6D5), AF647-conjugated	Biolegend	Cat # 115522
Anti-Mouse CD31 (Clone 390), purified	Biolegend	Cat # 102401
Anti-Mouse CD40 (Clone FGK45.5), purified	BioXCell	Cat # BE 0016-2
Anti-Mouse CD45 (Clone 30-F11), biotinylated	Biolegend	Cat # 103104
Anti-Mouse CD45 (Clone 30-F11), PE-Cy7-conjugated	Biolegend	Cat # 103114
Anti-Mouse CD62L (Clone MEL-14), PE-Cy7-conjugated	Thermo Fisher	Cat # 25-0621-82
Anti-Mouse CD63 (CloneNVG-2), biotinylated	Biolegend	Cat #143918
Anti-Mouse CD69 (Clone H1.2F3), PerCP/Cy5.5-conjugated	Biolegend	Cat # 104522
Anti-Mouse CD81 (clone Eat-2), purified	eBioscience	Cat # 14-0811-85
Anti-Mouse CD81 (CloneEat-2), biotinylated	Biolegend	Cat # 104903
Anti-Mouse CD90.1 (Clone HIS51), APC-conjugated	Thermo Fisher	Cat # 17-0900-82
Anti-Mouse CD163 (Clone S15049I), purified	Biolegend	Cat #155302
Anti-Mouse CD169 (Clone 3D6.112), purified	Biolegend	Cat # 142402
Anti-Mouse Caspase 3 (Clone), NucView 405-conjugated	Biotium	Cat # 10407
Anti-Mouse CD366 (Clone B8.2C12), PerCP-Cy5.5-conjugated	Biolegend	Cat # 134012
Anti-Mouse CD279 (Clone 29F.1A12) PE-Cy7-conjugated	Biolegend	Cat # 135216
Anti-Mouse CD223 (Clone C9B7W) BUV-737-conjugated	BD	Cat # 741820
Anti-Mouse F4/80 (Clone BM8), purified	eBioscience	Cat # 14-4801-85
Anti-Mouse FDC (Clone FDC-M1), purified	BD	Cat # 551320
Anti-Mouse Granzme B (Clone GB11), Pacific blue-conjugated	Biolegend	Cat # 515408
Anti-Mouse H-2Kb bound to SIINFEKL (Clone 25-D1.16), PE/Dazzle 594-conjugated	Biolegend	Cat # 141612
Anti-Mouse IFN-γ (Clone XMG1.2), PerCP-Cy5.5-conjugated	BD	Cat # 560660
Anti-Mouse Vα2 TCR (Clone B20.1), BV605-conjugated	BD	Cat # 562944
Anti-Mouse Pan-Keratin (Clone 11), biotinylated	Cell Signaling	Cat # 4279S
Anti-Mouse/Human CD63 mouse monoclonal IgG (clone LAMP3/2788), purified	NeoBiotechnologies	Cat # 967-MSM8-P1
Anti-Mouse / human desmin, goat polyclonal IgG, purified	R&D Systems	Cat# AF3844
Anti-Human CD1c (Clone AD5-8E7), FITC-conjugated	Miltenyi Biotec	Cat # 120-000-888
Anti-Human CD3 (Clone SP34-2), FITC-conjugated	Biolegend	Cat # 557705
Anti-Human CD3 (Clone SK7), APC/Cy7-conjugated	Biolegend	Cat # 344818
Anti-Human CD4 (Clone RPA-T4), APC-Cy7-conjugated	BD Pharmingen	Cat # 557871
Anti-Human CD8 (Clone SK1), BV421-conjugated	Biolegend	Cat # 344748
Anti-Human CD19 (HIB19), PerCP/Cy5.5-conjugated	Biolegend	Cat # 302229
Anti-Human CD19 (Clone HIB19), APC-conjugated	Biolegend	Cat # 302212
Anti-Human CD33 (Clone P67.6), BV605-conjugated	Biolegend	Cat # 366612
Anti-Human CD45 (Clone HI30), BV605-conjugated	Biolegend	Cat # 304042
Anti-Human CD45 (Clone HI30), BUV737-conjugated	BD	Cat # 748719

Anti-Human CD45 (Clone HI30), PE-conjugated	Biolegend	Cat # 304008
Anti-Human CD45 (Clone 30-F11), PE/Cy7-conjugated	Biolegend	Cat # 103114
Anti-Human CD141 (Clone AD5-14H12), APC-conjugated	Miltenyi Biotec	Cat # 130-090-907
Anti-Human CD163 (Clone GHI/61), purified	Biolegend	Cat # 33602
Anti-Human CD169 (Clone 7-239), purified	Biolegend	Cat # 346002
Anti-Human HLA-DR (Clone L243), Pacific blue-conjugated	Biolegend	Cat # 307624
Human TruStain FcX	Biolegend	Cat #422302
Anti-Grp94, rat monoclonal IgG2a (clone 9G10)	Enzo	Cat# ADI-SPA-850-D
Anti-GM130 (clone ARC0589)	Invitrogen	MA5-35107
Anti-Calnexin polyclonal	Invitrogen	PA5-34754
Anti-OVA, rabbit polyclonal IgG, purified	Abcam	Cat # 181688
Anti-OVA, mouse monoclonal IgG1, purified	Abcam	Cat # 17293
Rat IgG2a, Isotype Control (Clone RTK2758), biotynylated	Biolegend	Cat # 400503
Anti-Mouse IgG, AF488-conjugated	Thermo Fisher	Cat # A11029
Anti-Rabbit IgG, AF488-conjugated	Thermo Fisher	Cat # A21206
Anti-Rat IgG, AF488-conjugated	Thermo Fisher	Cat # A21208
Anti-Rat IgG, AF555-conjugated	Invitrogen	Cat # A21434
Anti-Rat IgG, AF647-conjugated	Thermo Fisher	Cat # A21247
Anti-Goat IgG, AF647-conjugated	Invitrogen	Cat # A21447
Anti-Armenian Hamster IgG, Cy3 conjugated	Jackson Immuno-Research	Cat # 127-165-160
Rat IgG2a (Clone eBR2a), purified	Thermo Fisher	Cat # 14-4321-85
Streptavidin, AF488-conjugated	Life Technologies	Cat # A32361
Streptavidin, AF647-conjugated	Thermo Fisher	Cat # S32357
Streptavidin, Cy3-conjugated	Jackson Immuno-Research	Cat # 016-160-084
Mouse monoclonal anti-calnexin (Clone GT1563)	Invitrogen	Cat # MA5-31501
Rabbit polyclonal anti-Tsg101	Invitrogen	Cat # PA5-82236
Rabbit monoclonal anti-CD63 (Clone SY21-02)	Novus Biotechne	Cat # NBP2-67425
Rabbit polyclonal anti-CD81	Invitrogen	Cat # PA5-114717
Donkey anti-mouse IgG, IRDye®680LT-conjugated	Li-Cor	Cat # 926-68022
Anti-Rabbit IgG, 6nm Colloidal Gold-conjugated	Jackson Immuno-Research	Cat # 111-195-144
Chemicals, Peptides, and Recombinant Proteins		
CellTracker^{TM} CM-Dil	Thermo Fisher	Cat # C7000
Vybrant CFDA SE Cell Tracer Kit	Thermo Fisher	Cat # V12883
eBioscience Cell Stimulation Cocktail (500x)	Invitrogen	Cat # 00-4970-93
Fetal Bovine Serum	Gemini	Cat # 100-106
Bovine Serum Albumin	Sigma-Aldrich	Cat # A2058-25G
Sodium Azide	Sigma-Aldrich	Cat # S2002
Sodium Bicarbonate	Fisher Scientific	Cat # BP328-500
Gold Conjugation Kit (20nm, 20OD)	Abcam	Cat # ab188215
Gold Conjugation Kit (10nm, 20OD)	Abcam	Cat # 201808
Glycine	J.T. Baker	Cat # 4059-00
RIPA Buffer	Sigma-Aldrich	Cat # R0278-50ml
Micro Tube 2ml, PP	Sarstedt	Cat # 72.694.006
Ficoll-Paque Plus	Cytiva	Cat # 17144002
Percoll	Sigma-Aldrich	Cat # P1644-500ml

Medium NCTC-109 (1x)	Gibco	Cat # 21340-039
Medium 199	Sigma-Aldrich	Cat # M9163-500ml
Dynabead Exosome – Streptavidin for Isolation/Detection	Thermo Fisher	Cat # 10608D
Anotop 10 Plus (0.02µm Filter)	Whatman	Cat # 6809-3002
ExoDisc-D20	LabSpinner	Cat # EX-D0020
Matrigel Matrix	Corning	Cat # 354234
Slide-A-Lyzer™ Dialysis Cassettes, 10K MWCO	Thermo Fisher	Cat # 66383
Millex® polyethersulfone syringe filter (.22µm pore size)	Sigma-Aldrich	Cat # SLGPR33RS
Horse Serum	Sigma-Aldrich	Cat # H0146-10ml
Intercept® Blocking Buffer	Li-Cor	Cat # 927-70001
DAPI	Thermo Fisher	Cat # D1306
Fixable Viability Dye eFluor780	eBioscience	Cat # 65-0865-14
Heparin (10,000 USP units per 10 ml)	Mylan Pharmaceuticals	N/A
Red Blood Cell Lysing Buffer Hibri-Max™	Sigma-Aldrich	Cat # R7757-100ML
Neomycin	Sigma-Aldrich	Cat # N6386
Polymyxin B	Sigma-Aldrich	Cat # P4932
Dimethyl Sulfoxide	Fisher Scientific	Cat # BP231-100
2-Methylbutane	Honeywell	Cat # M32631-1L
Vectabond	Vector	Cat # SP-1800
Goat Serum	Sigma-Aldrich	Cat # G9023-10ml
Paraformaldehyde	Sigma-Aldrich	Cat # P6148-500G
EDTA	Sigma-Aldrich	Cat # 1233508
Hepes Solution	Sigma-Aldrich	Cat # H3537-100ML
RPMI 1640	Corning	Cat # 15-040-CV
Dulbecco's modified Eagle's medium (DMEM)	Fisher	Cat # 11-965-118
Soluble OVA	Worthington	Cat # LS 003061
Poly I:C	Sigma-Aldrich	Cat # P1530
L-Glutamine-Penicillin-Streptomycin Solution	Sigma-Aldrich	Cat # G6784
2-Mercaptoethanol	Sigma-Aldrich	Cat # M7522
MEM Non-essential Amino Acid Solution	Sigma-Aldrich	Cat # M7145
Sodium Pyruvate Solution	Sigma-Aldrich	Cat # S8636-100ML
Normal PBS	Genesee Scientific	Cat # 25-507B
PBS BioPerformance certified, for molecular biology	Millipore-Sigma	Cat # P5493
OCT Tissue Plus	Fisher Scientific	Cat # 4585
Vector® TrueVIEW® Autofluorescence Quenching Kit	Vector Laboratories	Cat # SP-8400
Vectashield® vibrance mounting media	Vector Laboratories	Cat # H-1700
Glutaraldehyde	Energy Beam Sciences	Cat # T-GL-500
Toluidine Blue	Fisher Scientific	Cat # T161-25
Uranyl Acetate	Electron Microscopy Services	Cat # 22400
Polyvinylpyrrolidone	Sigma-Aldrich	Cat # PVP-10
Sucrose	Fisher Scientific	Cat # BP220-1
Sodium Carbonate	Fisher Scientific	Cat # S263-500
Methyl Cellulose	Fisher Scientific	Cat # M352-500
VivaCell 100	Satorius	Cat # VC1042
Optiprep Density Gradient Medium	Sigma-Aldrich	Cat # D1556-250
Q5 High-Fidelity 2x Master Mix	New England BioLabs	Cat # M0492s
BD Microtainer® with K_2EDTA	Beckton Dickinson	Cat # 365974
Liberase™ DH Research Grade	Millipore-Sigma	Cat # 5401054001

DNAse I	Roche	Cat # 10104159001
Halt™ Protease & Phosphatase Inhibitor Cocktail	Thermo Fisher	Cat # 78440
Dynabeas™ M-280 Sheep anti-rabbit IgG	Invitrogen	Cat # 11203D
CD34 MicroBead Kit (Human)	Miltenyi	Cat # 130-046-702
Dynabeads Untouched Mouse CD4 Cells	Thermo Fisher	Cat # 11415D
Dynabeads Untouched Mouse CD8 Cells	Thermo Fisher	Cat # 11417D
PureProteome™ Magnetic Stand	Millipore Sigma	Cat # LSKMAGS08
4X Laemmli sample buffer	Bio Rad	Cat # 1610747
Mini-Protean TGX Gels	Bio Rad	Cat # 4561033
Immun-Blot® LF PVDF membranes and filter papers	Bio Rad	Cat # 162-0261
Tween 20	Bio Rad	Cat # 1706531
Avidin/biotin blocking kit	Fisher Scientific	Cat # NC9406552
Experimental Models: Organisms/Strains		
Mouse: BALB/cAnNCr	Charles River	Cat # 555
Mouse: C57BL/6NCr	Charles River	Cat # 556
Mouse: NSG-SGM3	The Jackson Laboratoy	Cat # 013062
Mouse: Act-mOVA	The Jackson Laboratoy	Cat # 005145
Mouse: Nuclear-BFP / mScarlet-CD63^{LSL} B6	In house generated	N/A
Mouse Rab27a^{KO}	S.D. Catz. The Scripps Research Institute, La Jolla, CA. USA.	N/A
Mouse B6.C-Tg (CMV-Cre)1Cgn/J	The Jackson Laboratoy	Cat # 006054
Mouse B6 Exomap1	S.J. Gould. Johns Hopkins University, Baltimore, MD. USA.	N/A
Mouse B6 Rag1$^{-/-}$ Thy1.1^{+} OT-I	F. Lakkis, University of Pittsburgh, Pittsburgh, PA. USA.	N/A
Mouse B6 Thy1.1^{+} FIR OT-II	G. Camirand. University of Pittsburgh, Pittsburgh, PA. USA	N/A
Oligonucleotides		
Rosa26 F06	Integrated DNA Technologies	5’-CTCGGCTAGGTAGGGGATCG-3’
Rosa26 R06	Integrated DNA Technologies	5’-TGGACTACTGCGCCCTACA-3’
hPacs1 F1	Integrated DNA Technologies	5’-TTGTGGTTTGTCCAAACTCAT-3’
mTagBFP R1	Integrated DNA Technologies	5’-TTCTTCTGCATAACTGGTCCGT-3’
mTagBFP F1	Integrated DNA Technologies	5’-GCCTTATGAGGGAACCCAGAC-3’

CD63 R1	Integrated DNA Technologies	5'-CACCCACTGCAATGATGACCA-3'
CD63 F1	Integrated DNA Technologies	5'-TTGGTGTAGCGGTTCAGGTTGT-3'
Rosa26 R05	Integrated DNA Technologies	5'-CAGAAGACTCCCGCCCATCT-3'
Rosa26 F02	Integrated DNA Technologies	5'-CGTTTCCGACTTGAGTTGCC-3'
Rosa26 R02	Integrated DNA Technologies	5'-ACTCGGGTGAGCATGTCTTT-3'
Software and Algorithms		
Metamorph 7.7.50 software	Molecular Devices	https://www.moleculardevices.com/products/cellular-imaging-systems/acquisition-and-analysis-software/metamorph-microscopy#gref
NTA Software 2.0	Malvern Panalytical	https://www.malvernpanalytical.com/en/support/product-support/software/NanoSight-NTA-software-update-v3-2
IDEAS v6.2. software	Andritz Group	https://www.andritz.com/resource/blob/15094/317004ed1e6f9cda9efe0a600f397037/aa-ideas-v600-user-manual-data.pdf
FlowJo v.10	FlowJo, LLC	https://www.flowjo.com/
GraphPad Prism v7	GraphPad	https://www.graphpad.com/
Adobe Photoshop v25.11.0	Adobe	https://www.adobe.com
