# Supplementary material for: Fetoplacental extracellular vesicles deliver conceptus-derived antigens to maternal secondary lymphoid tissues for immune recognition
Source: JCI Insight. 2025 May 22;10(10):e186335. doi: 10.1172/jci.insight.186335 (PMC12128977; doi:10.1172/jci.insight.186335)
Supplement: Unedited blot and gel images [file jciinsight-10-186335-s165.pdf]

# Full unedited gels (Ms. 186335)

Bands used for figures:

Figure 4B

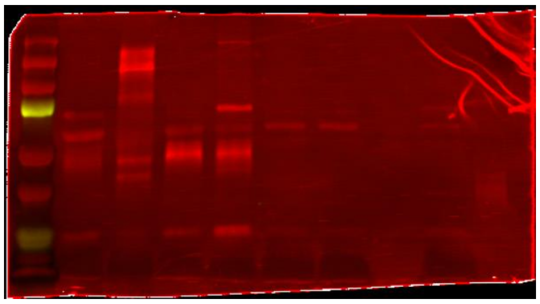

mOVA →

Figure 4B

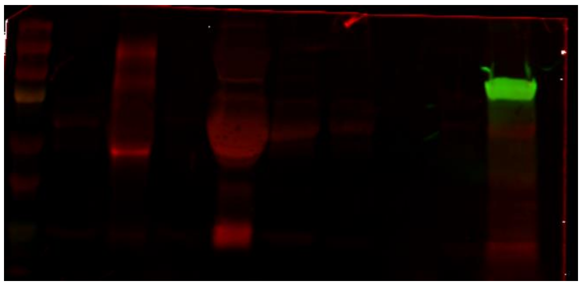

Gp96 → (green)

Figure 4B

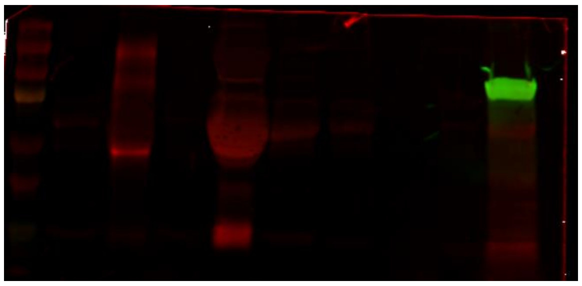

CD63 →

Figure 4B

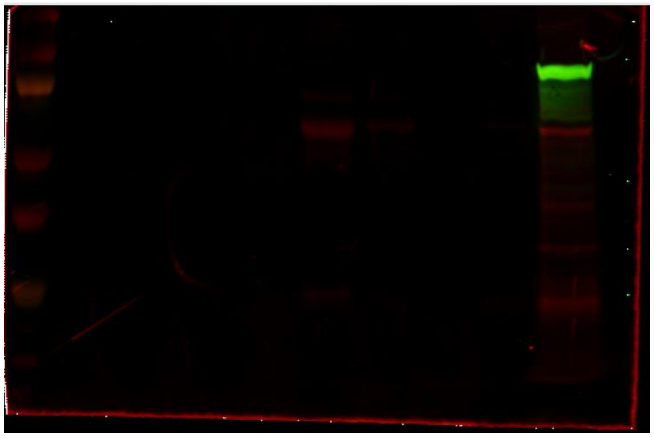

CD81 →

Figure 4C

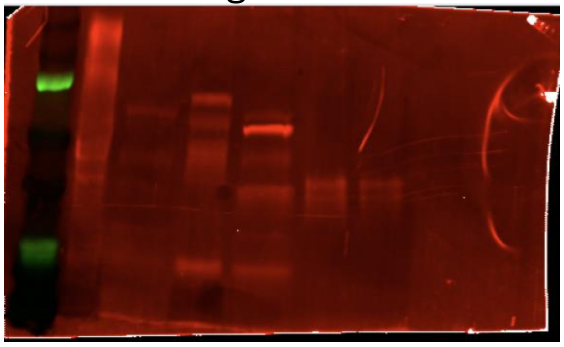

mOVA →

Figure 4D

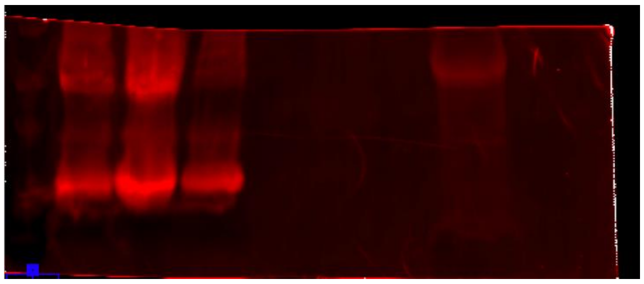

CD81 →

Bands used for figures:

Figure 4D

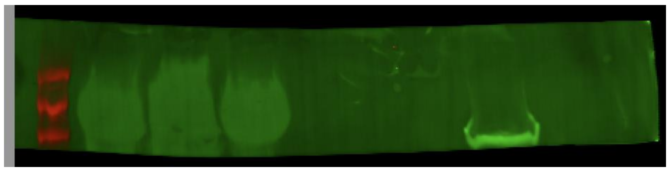

Gp96 →

Figure 4D

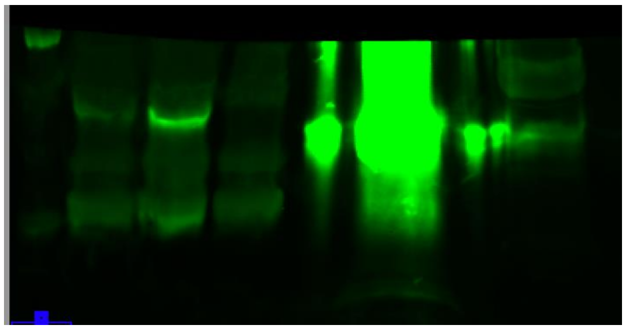

mOVA →

Figure 5D

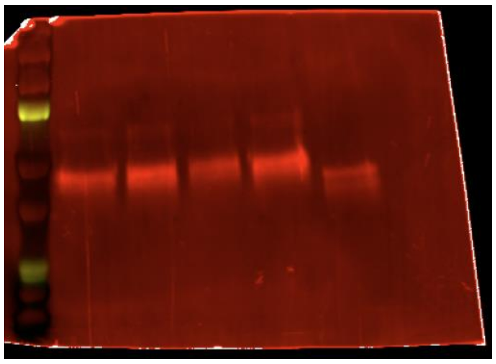

mOVA →

Figure 5D

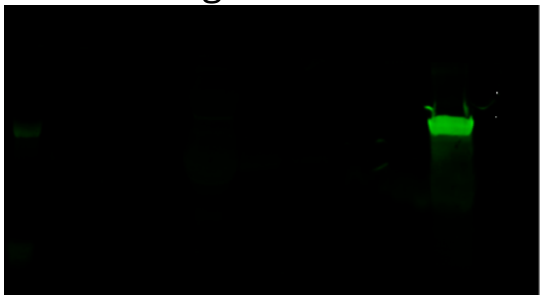

Gp96 →

Figure 5D

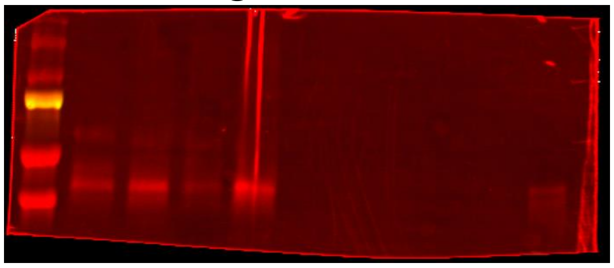

mOVA →

# Full unedited gels (Ms. 186335)

Bands used for figures:

Figure 8A

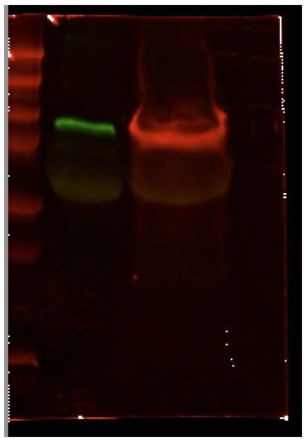

CD63 (green) →
Calnexin (red)

Figure 8A

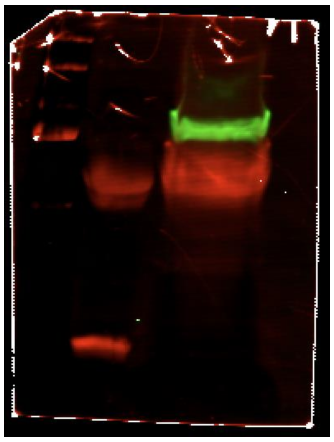

Hsp90B1 (green) →

CD81 (red) →

Figure 8A

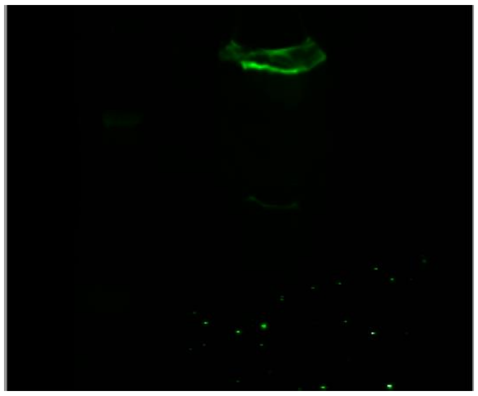

GM130 →

Figure 8A

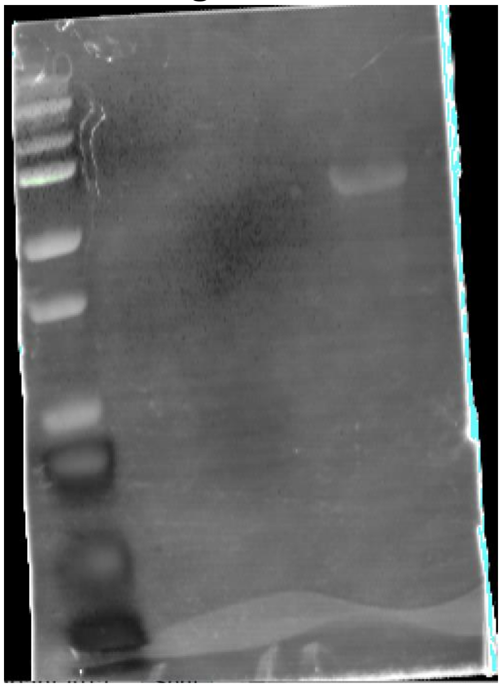

Tsg101→
